# Structural and functional insights into *Pseudomonas aeruginosa* ApaH, a diadenosine tetraphosphatase crucial for bacterial virulence

**DOI:** 10.1002/pro.70781

**Published:** 2026-08-25

**Authors:** Gianluca Pistoia, Matteo Cervoni, Flavia Catalano, Francesca Troilo, Francesca Guidi, Eleonora Comparini, Giuseppina Mignogna, Carlo Travaglini‐Allocatelli, Alessandro Giuffrè, Antonio Coluccia, Francesco Imperi, Adele Di Matteo, Giorgio Giardina

**Affiliations:** ^1^ Department of Biochemical Sciences “A. Rossi Fanelli” Sapienza University of Rome Rome Italy; ^2^ Department of Science University Roma Tre Rome Italy; ^3^ Institute of Molecular Biology and Pathology National Research Council Rome Italy; ^4^ Department of Drug Chemistry and Technologies Sapienza University of Rome Rome Italy; ^5^ LIFE Institute for Research and Health Care Santa Lucia IRCCS Rome Italy

**Keywords:** ApaH, cystic fibrosis, diadenosine tetraphosphate, dinucleotides, Gram‐negative bacteria, RNA‐capping, swarming, virulence

## Abstract

Infections by *Pseudomonas aeruginosa* are a major cause of severe morbidity and mortality in immunocompromised patients and people with cystic fibrosis, largely due to the pathogen's ability to resist antibiotic treatment and to deploy multiple virulence strategies that promote persistence in the host. We have recently uncovered that the signaling molecule diadenosine tetraphosphate (Ap4A) acts as a crucial regulator of *P. aeruginosa* virulence. Specifically, deletion of the Ap4A‐degrading enzyme, diadenosine tetraphosphatase (ApaH), dramatically reduces the expression of key virulence factors. The structural and molecular properties of *P. aeruginosa* ApaH (*Pa*ApaH) remain uncharacterized. Here, we present an integrated biochemical, structural, computational, and phenotypic characterization of *Pa*ApaH. We define the molecular determinants of its manganese‐dependent Ap4A hydrolytic mechanism and establish a direct functional link between *Pa*ApaH catalytic activity and virulence phenotypes *in vivo*. Together with the absence of ApaH homologs in eukaryotes, these findings place *Pa*ApaH as an attractive and selective target for antivirulence therapeutic strategies against *P. aeruginosa* infections.

## 1 INTRODUCTION

*Pseudomonas aeruginosa* (*Pa*) is a Gram‐negative bacterium extensively studied for its pathogenicity, particularly in the context of opportunistic infections in hospitalized and immunocompromised patients, and people with cystic fibrosis (Reynolds & Kollef, 2021). This organism is known for its metabolic versatility, ability to survive in diverse environments, large arsenal of virulence factors, and intrinsic and acquired resistance to multiple antibiotics (Jurado‐Martín et al., 2021; Moradali et al., 2017; Rossi et al., 2021). Recent studies have shown that in *Pa*, the deletion of the *apaH* gene coding for the diadenosine tetraphosphatase (or diadenosine tetraphosphate hydrolase) (ApaH) leads to a strong downregulation of virulence genes and reduced infectivity in various infection models, including a mouse model of lung infection (Cervoni et al., 2024). ApaH is responsible for the hydrolysis of diadenosine tetraphosphate (Ap4A).

Ap4A consists of two adenosine molecules linked by a chain of four phosphate groups, joined via phosphoester bonds to their respective 5′‐hydroxyl groups. Since its discovery in 1966 (Zamecnik et al., 1966), it has been identified across all domains of life (Kisselev et al., 1998; Varshavsky, 1983), and decades of research have sought to elucidate its role in both eukaryotic and prokaryotic organisms. However, despite its ubiquitous presence, the physiological role of Ap4A remains incompletely understood. Indeed, it is still debated whether Ap4A is merely a byproduct of cellular damage (Despotović et al., 2017) or serves a regulatory role as an alarmone, a hypothesis that is currently more widely supported (Ferguson et al., 2020; Young & Wang, 2024; Zegarra et al., 2023).

In bacteria, Ap4A exerts pleiotropic effects. Apart from its involvement in the expression of virulence traits in *Pa* (Cervoni et al., 2024), in other species, it has been associated with cell division (Nishimura et al., 1997), motility (Farr et al., 1989), responses to heat and oxidative stress (Johnstone & Farr, 1991; Lundin et al., 2003), biofilm formation (Monds et al., 2010), tolerance to aminoglycoside antibiotics (Ji et al., 2019) as well as adhesion and invasion of mammalian cells in *Salmonella enterica* (Ismail et al., 2003; Lee et al., 1983).

To clarify the mode of action of Ap4A, recent studies have focused on identifying proteins that directly bind this molecule. These studies revealed that Ap4A may act as a signaling molecule through interaction with proteins involved in proteostasis such as the chaperones DnaK, ClpB, and GroEL, in central metabolism, and in nucleotide biosynthesis (Giammarinaro et al., 2022; Guo et al., 2011; Krüger et al., 2021; Young & Wang, 2024). In *Bacillus subtilis*, Ap4A has been shown to be involved also in the regulation of protein acetylation (Zheng et al., 2026).

Ap4A is generally synthesized as a byproduct of some aminoacyl‐tRNA synthetases (Brevet et al., 1989), and its levels increase in response to various types of stress (Ferguson et al., 2020). Cellular homeostasis of Ap4A is maintained by a variety of hydrolases and/or phosphorylases (Young & Wang, 2024). In bacteria, Ap4A hydrolysis is mediated by different enzyme classes that differ in cofactor requirements and catalytic mechanism, providing either asymmetrical (to ATP and AMP) or symmetrical (to two ADP) cleavage of the substrate. Enzymes from the Nudix or the histidine triad (HIT) families cleave Ap4A asymmetrically (Guranowski, 2000; McLennan, 2006), while those belonging to the families of YqeK and ApaH catalyze a symmetrical cleavage in Gram‐positive and Gram‐negative bacteria, respectively (Ferguson et al., 2020; Guranowski et al., 1983; Minazzato et al., 2020; Nuthanakanti et al., 2025; Ueda et al., 2024). Notably, recent studies showed that *Escherichia coli* ApaH, together with the 5′ pyrophosphohydrolase RppH, plays a regulatory role on mRNA degradation and transcription by removing the 5′ Np(n)N‐cap, thus regulating mRNA stability and degradation (Hudeček et al., 2020; Luciano et al., 2019; Luciano & Belasco, 2020; Nuthanakanti et al., 2025), a finding possibly explaining the observed pleiotropic effects of Ap4A in different bacterial species and our previous results on the *Pa* Δ*apaH* mutant showing increased levels of Ap4A and reduced virulence (Cervoni et al., 2024).

Importantly, ApaH has no homologs in mammalian organisms, as in eukaryotes Ap4A hydrolases typically belong to the Nudix family (Andreeva & Kutuzov, 2004; Ferguson et al., 2020). This makes ApaH from *Pa* (*Pa*ApaH) a promising therapeutic target for the development of antivirulence strategies against *Pa* infection.

In this study, we provide a comprehensive functional and structural characterization of the enzyme followed by an in vivo analysis of catalytically impaired *Pa*ApaH mutants. Using an integrated approach combining biochemical assays, structural biology, and bacterial phenotyping, we identify *Pa*ApaH as a symmetrical hydrolase capable of degrading Ap4A, Ap3A, Ap5A, and Gp4G in the presence of Mn^2+^ ions. We report the high‐resolution crystal structures of the Mn^2+^ and Mg^2+^‐bound *Pa*ApaH together with the structure of the ADP‐bound enzyme. Structural data, combined with molecular docking studies, enabled us to identify key residues involved in substrate binding and catalysis. Crucially, for two mutants, we observed a direct correlation between *in* *vitro* catalytic activity and *in* *vivo* regulation of virulence traits in *Pa*. These findings not only deepen our understanding of diadenosine polyphosphate metabolism in bacteria but also provide a molecular foundation for the development of novel antivirulence therapies targeting *Pa*.

## 2 RESULTS

### 2.1 *Pa*ApaH is a Mn‐dependent symmetrical dinucleotide hydrolase

**FIGURE 1:**
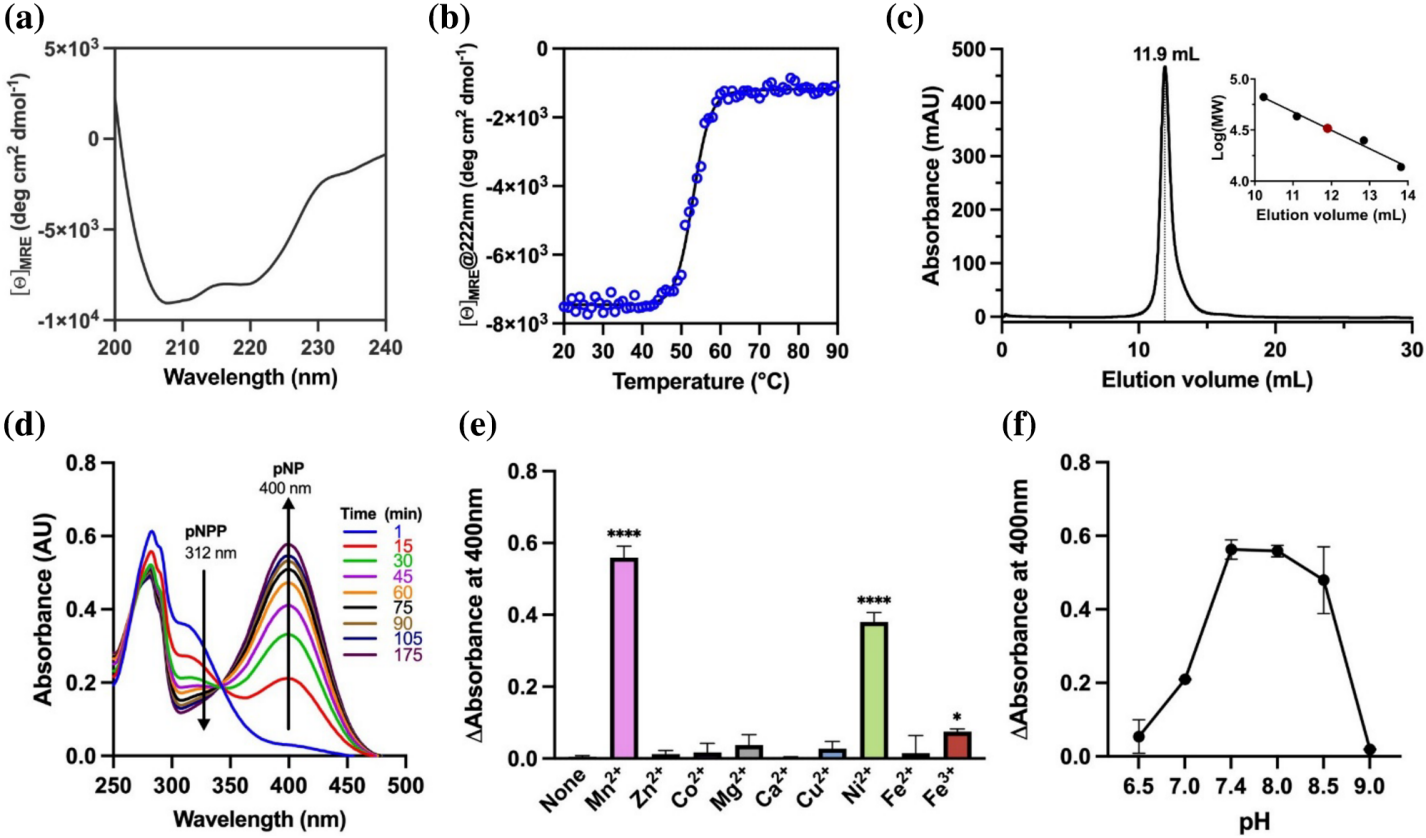
Biochemical and preliminary functional characterization of recombinant *Pseudomonas aeruginosa* diadenosine tetraphosphatase (*Pa*ApaH). (a) Far‐UV circular dichroism spectrum of *Pa*ApaH at 20°C in 20 mM Tris–HCl (pH 7.4), 100 mM NaCl. (b) Thermal denaturation profile of *Pa*ApaH acquired by measuring the protein ellipticity at 222 nm; an apparent melting temperature (*T*_m,app_) of ∽53.0°C was obtained by fitting the data to a sigmoidal curve. (c) Size‐exclusion chromatography profile of *Pa*ApaH in 20 mM Tris–HCl (pH 7.4), 100 mM NaCl. Inset: Calibration curve using commercial protein standards (black full circles: Protein standards; red full circle: *Pa*ApaH). (d) Time‐resolved UV–vis absorption spectra acquired during *p*‐nitrophenyl phosphate (pNPP) (50 μM) hydrolysis by *Pa*ApaH (10 μM) at 25°C in 20 mM Tris–HCl (pH 7.4), 100 mM NaCl, 2 mM MnCl_2_. (e) Effect of various metal ions on *Pa*ApaH (1 μM) phosphatase activity toward pNPP (100 μM): ΔAbsorbance measured at 400 nm after 3 h of incubation in 20 mM Tris–HCl (pH 7.4), 100 mM NaCl, and 1 mM of different metal ions. Statistical significance was assessed by the one‐way analysis of variance method (*p < 0.05; ****p < 0.0001). (f) Effect of pH on *Pa*ApaH hydrolase activity toward pNPP measured as in panel (d) with 1 mM MnCl_2_ and varying the buffer systems. In both panels (e) and (f) error bars represent standard deviation of three independent reactions. pNP, *p*‐nitrophenol.

*Pa*ApaH was expressed in *E. coli* and purified to homogeneity, with a yield of ~10 mg L^−1^ of culture. The isolated recombinant protein was shown by far‐UV circular dichroism (CD) to predominantly have α/β secondary structure (Figure 1a) and to be well folded, as revealed by the cooperative, yet irreversible (not shown) transition observed upon thermal denaturation (Figure 1b). Size‐exclusion chromatography (SEC) displayed a monodisperse elution profile, with an elution volume corresponding to an apparent molecular weight of ~32.8 kDa (Figure 1c), consistent with a monomeric state of the protein in solution (32.3 kDa).

To confirm that the purified *Pa*ApaH was active and to characterize the effect of different metal ions and pH on its catalytic activity, we first monitored spectrophotometrically the conversion of *p*‐nitrophenyl phosphate (pNPP) used as a model substrate into *p*‐nitrophenol (pNP) (Figure 1d). Although pNPP is not the physiological substrate of ApaH, this pseudo‐substrate is routinely used to monitor phosphatase activity. With this assay, reaction kinetics can be followed in real time by measuring the absorbance of the product, pNP, at 400 nm using a plate reader. This approach allows a convenient preliminary screening of many different reaction conditions. *Pa*ApaH phosphatase activity is significantly enhanced in the presence of Mn^2+^ or Ni^2+^, with Mn^2+^ being the most effective ion (Figure 1e), in agreement with previous findings for ApaH homologs from *Myxococcus xanthus* (Sasaki et al., 2014). The pH‐dependent phosphatase activity profile, determined in the presence of Mn^2+^, revealed a catalytic optimum between pH 7.4 and 8.0 (Figure 1f).

**FIGURE 2:**
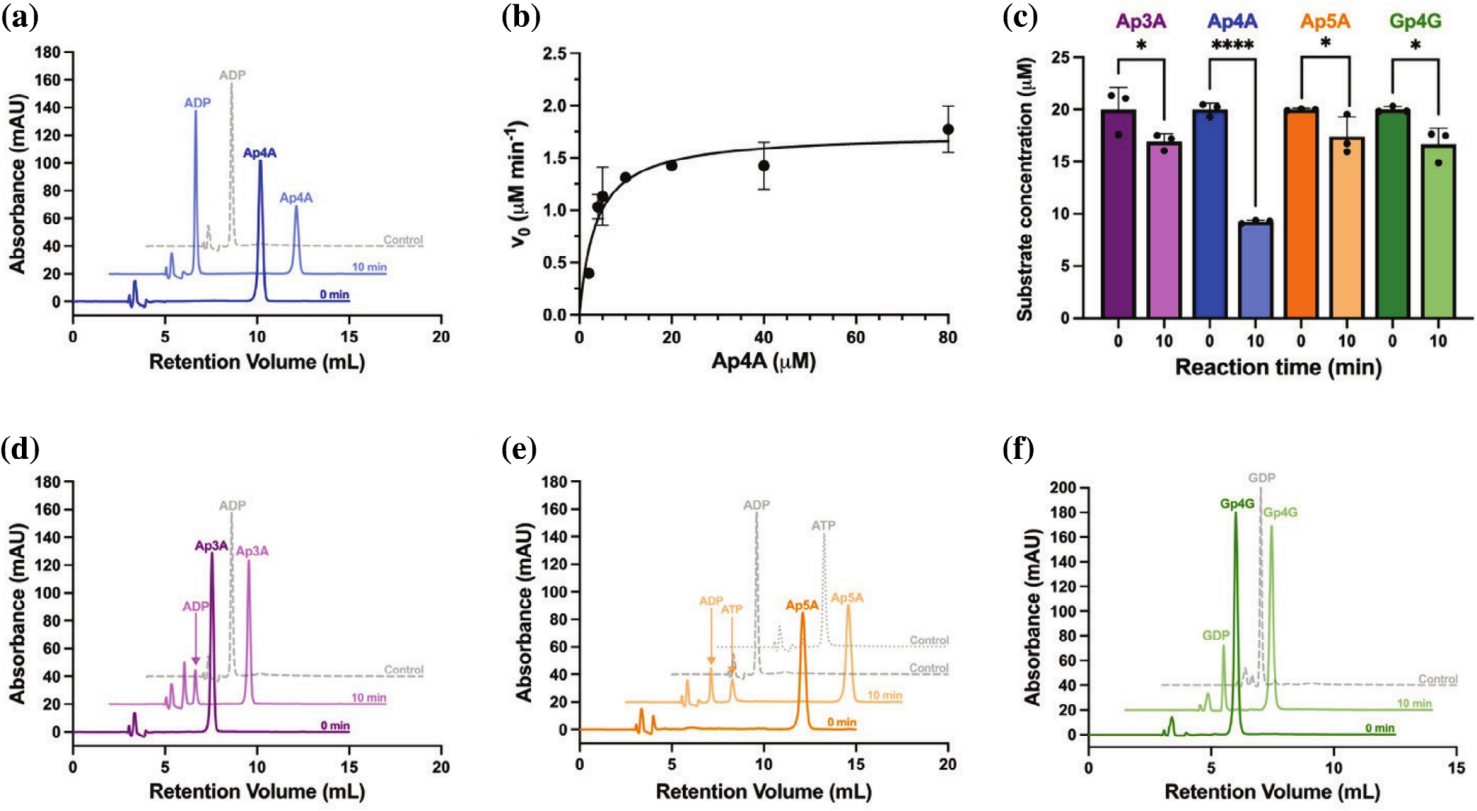
Kinetic analysis and substrate specificity of *Pseudomonas aeruginosa* diadenosine tetraphosphatase (*Pa*ApaH). (a) Hydrolysis of diadenosine tetraphosphate (Ap4A) by *Pa*ApaH followed by ion‐pair reversed‐phase high‐performance liquid chromatography. Chromatograms of the reaction mixture (5 nM *Pa*ApaH in 20 mM Tris–HCl pH 7.4, 100 mM NaCl, 2 mM MnCl_2_ and 20 μM Ap4A) before (dark blue) or after (light blue) 10 min‐incubation at 20°C. The gray dashed line shows elution of the ADP standard (20 μM). (b) Michaelis–Menten plot of *Pa*ApaH (5 nM) hydrolase activity measured with Ap4A as substrate in the same condition as panel (a). Initial velocities were determined at varying Ap4A concentrations (mean ± standard deviation [SD]; *n* = 3). (c) Substrate specificity of *Pa*ApaH. Histograms represent substrates concentration before (dark color) and after (light color) 10 min‐incubation with *Pa*ApaH in the same conditions as panel (a) (mean ± SD; *n* = 3). Unpaired Student's *t*‐test was used to assess statistical significance (*p < 0.05; ****p < 0.001). (d–f) Chromatograms of the reaction mixture before (dark spectra) or after (light spectra) 10 min‐incubation at 20°C with Ap3A, Ap5A, or Gp4G in place of Ap4A, respectively. Reactions were carried out in the same conditions as panel (a). The gray dashed line shows elution of the nucleotide standards.

We next measured by Ion‐Pair Reversed‐Phase chromatography (IP‐RP‐HPLC) the catalytic activity of *Pa*ApaH with the physiological substrate Ap4A; experiments were carried out in 20 mM Tris–HCl (pH 7.4), 100 mM NaCl, 2 mM MnCl_2_, in the presence of 5 nM enzyme and 20 μM Ap4A at 20°C. Under these conditions, that is, in the presence of Mn^2+^, *Pa*ApaH catalyzed the symmetrical cleavage of one molecule of Ap4A into two molecules of ADP (Figure 2a). Initial rate measurements performed at varying Ap4A concentrations showed that the reaction followed Michaelis–Menten kinetics, yielding a *K*_M_ of 3.6 ± 0.9 μM and a *V*_max_ of 1.7 ± 0.1 μM min^−1^ with a turnover number (*K*_cat_) of 5.7 ± 0.3 s^−1^ (Figure 2b). No activity was detected when Mg^2+^ (as MgCl_2_) was provided in place of Mn^2+^ as the divalent cation in the tested conditions (data not shown), consistently with what observed with pNPP as substrate (Figure 1e).

Furthermore, we investigated the activity of *Pa*ApaH toward other dinucleotides such as Ap3A, Ap5A, and Gp4G. Substrate consumption was assessed by measuring the residual amount of each compound (initial concentration 20 μM) after 10 min‐incubation at 20°C with 5 nM *Pa*ApaH in 20 mM Tris–HCl (pH 7.4), 100 mM NaCl, and 2 mM MnCl_2_. In these conditions, with all tested dinucleotides, *Pa*ApaH exhibited lower hydrolytic activity as compared to Ap4A (Figure 2c). Importantly, the reaction products of Ap3A, Ap5A, and Gp4G hydrolysis were identified as AMP + ADP, ADP + ATP, and two GDP molecules, respectively (Figure 2d), highlighting that *Pa*ApaH unambiguously cleaves the polyphosphate chain at the β‐position, with a preference for A over G. No activity was detected with either A(G)DP or A(G)TP in the same conditions, confirming that *Pa*ApaH requires a dinucleotide scaffold for catalysis (Figure S1).

### 2.2 A bimetallic center and an additional structural motif are key structural elements for *Pa* ApaH activity

The structures of *Pa*ApaH in complex with Mn^2+^ (*Pa*ApaH–Mn), Mg^2+^ (*Pa*ApaH–Mg), or ADP in the presence of Mg^2+^ (*Pa*ApaH–Mg–ADP) were solved at resolution below 1.9 Å (Table 1). Superposition of the three structures reveals that, overall, they are highly similar root‐mean‐square deviation (RMSD), across 266 pruned atoms of 0.27 Å and of 0.39 Å between *Pa*ApaH–Mn and *Pa*ApaH–Mg or *Pa*ApaH–Mn and *Pa*ApaH–Mg–ADP, respectively); thus, the *Pa*ApaH–Mn structure was taken as the reference for structure description and comparison.

**TABLE 1:** Crystallization, data collection, model building, and refinement statistics.

	*Pa*ApaH–Mg	*Pa*ApaH–Mn	*Pa*ApaH–Mg–ADP
PDB id	9T9N	9T9O	9TA7
Data collection			
Beamline	ESRF‐ID23‐2	ESRF‐ID23‐2	Elettra‐XRD2
Wavelength (Å)	0.873	0.873	1.000
Resolution range (Å)	61.55–1.89 (1.93–1.89)	61.41–1.75 (1.78–1.75)	42.53–1.55 (1.57–1.55)
Space group	P 21 21 21	P 21 21 21	P 21 21 21
Unit cell (Å, °)	76.33 76.93 102.62 90 90 90	74.87 77.58 100.51 90 90 90	75.32 76.38 102.28 90 90 90
Unique reflections	48,729 (2806)	59,655 (2659)	81,468 (2916)
Multiplicity	6.8 (6.9)	10.6 (10.7)	12.1 (12.3)
Completeness (%)	99.57 (98.21)	99.89 (99.85)	94.56 (99.45)
Mean I/sigma(I)	11.8 (1.1)	9.3 (2.1)	21.6 (2.0)
Wilson *B*‐factor	34.6	18.4	22.68
R‐merge	0.082 (1.729)	0.153 (1.100)	0.059 (1.439)
CC1/2 (%)	99.9 (52.8)	99.7 (77.7)	100.0 (80.0)
Model building and refinement			
Reflections used in refinement	48,729 (2806)	59,655 (2659)	81,468 (2916)
Reflections used for R‐free	2331 (152)	3074 (155)	4038 (133)
R‐work	0.206 (0.356)	0.183 (0.259)	0.186 (0.299)
R‐free	0.241 (0.403)	0.216 (0.310)	0.213 (0.302)
Number of non‐hydrogen atoms	4432	4606	4672
Macromolecules	4191	4212	4282
Ligands	4	10	60
Solvent	237	384	330
Protein residues	534	533	549
RMSD (bonds, Å)	0.008	0.008	0.006
RMSD (angles, °)	1.02	0.96	1.05
Ramachandran favored (%)	95.06	96.19	95.96
Ramachandran allowed (%)	4.18	3.43	4.04
Ramachandran outliers (%)	0.76	0.38	0.00
Average *B*‐factor	45.0	19.0	34.22
Macromolecules	45.97	19.30	33.96
Ligands	40.49	17.35	35.88
Solvent	45.35	26.46	37.24

*Note*: Statistics for the highest‐resolution shell are shown in parentheses.

Abbreviations: ESRF, European Synchrotron Radiation Facility; *Pa*ApaH, *Pseudomonas aeruginosa* diadenosine tetraphosphatase; PDB, Protein Data Bank; RMSD, root‐mean‐square deviation.

**FIGURE 3:**
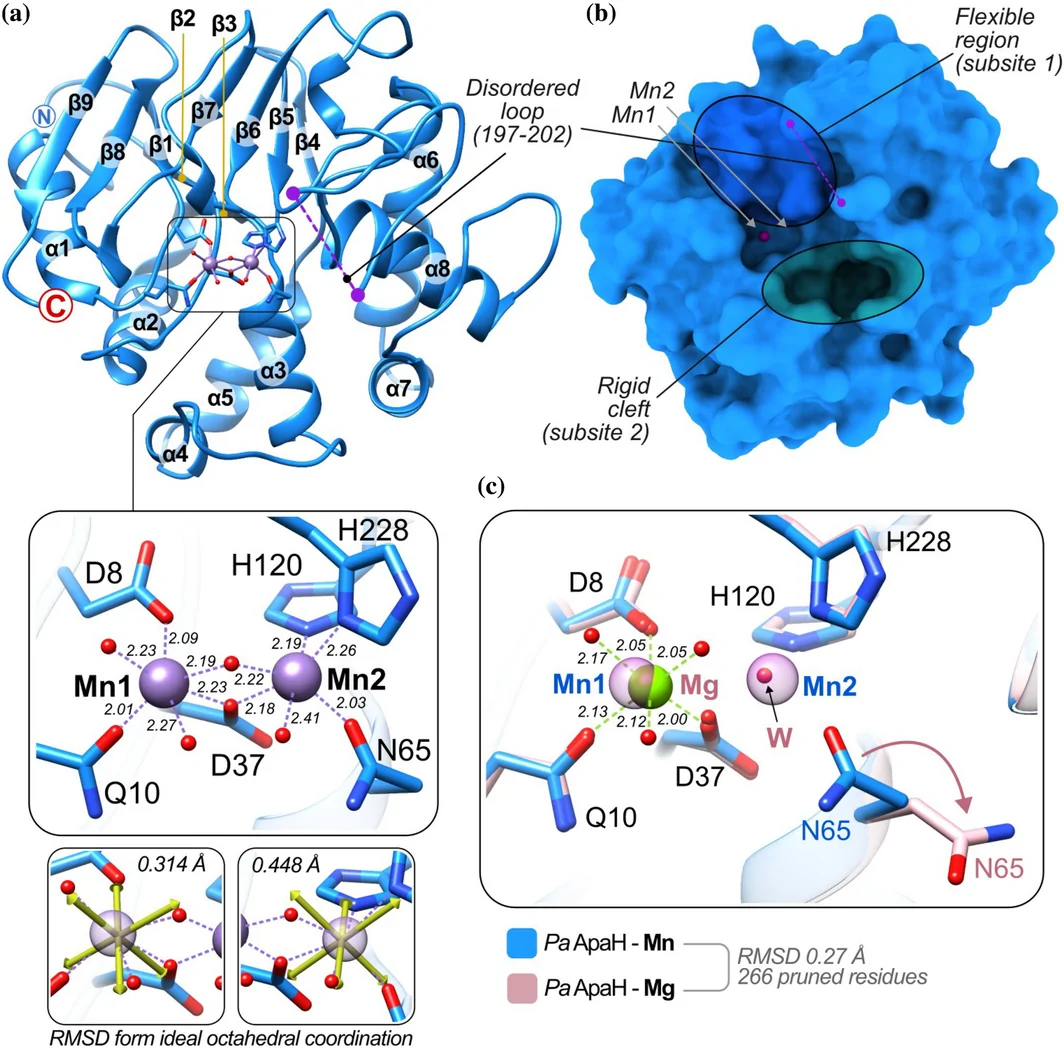
Structural features of *Pseudomonas aeruginosa* diadenosine tetraphosphatase (*Pa*ApaH). (a) Ribbon representation of *Pa*ApaH–Mn highlighting the secondary structure and the binuclear metal center (Mn^2+^ ions shown as purple spheres). The position of the N‐ and C‐terminus is indicated by a blue N and a red C, respectively. Each secondary structure element is numbered sequentially starting from the N‐terminus. Zoom: Close‐up of the binuclear Mn^2+^ site highlighting the residues involved in metal coordination. The coordination distances (dashed gray lines) for each ligand are shown in Å. Insets: Superposition of each Mn^2+^ coordination sphere onto an ideal octahedral geometry (solid yellow arrows). The ideal position for the Mn ligands is represented by the end of each arrow. The real ligands show small deviation from the ideal geometry. The corresponding root‐mean‐square deviation (RMSD) values (in Å) are shown to indicate the degree of distortion for each Mn ion. (b) Molecular surface representation of *Pa*ApaH–Mn showing the position of the flexible region (subsite 1) near the disordered loop (residues 197–202, the purple dashed lines indicate the residues that are connected by the disordered residues) and that of the rigid cleft (subsite 2) with respect to the active site. (c) Structural superposition of the active site of *Pa*ApaH structures in complex with Mn^2+^ (blue stick and purple semitransparent Mn ions) and Mg^2+^ (pink sticks and green Mg ion). The coordination spheres for Mn1 and Mg are conserved in both structures, whereas in the structure of *Pa*ApaH–Mg the Mn2 is replaced by a water molecule (W), with N65 that is flipped toward the solvent. The coordination distances (dashed green lines) are shown in Å only for the Mg^2+^ ion. The RMSD for the superposition of the two structures is indicated below the panel.

The overall architecture of *Pa*ApaH is shown in Figure 3a. The protein adopts a globular fold composed of eight α‐helices and nine β‐strands, stabilized by two disulfide bonds between C18–C267 and C248–C296. The β‐strands form two mixed β‐sheets that face each other. The β‐sandwich core is flanked on three sides by α‐helices and several loops. The active site is positioned within a groove formed by helices α2 and α3 and connecting loops. It hosts a binuclear center composed of two Mn^2+^ ions separated by 3.48 Å and arranged in a slightly distorted octahedral coordination geometry (Figure 3a). Mn1 is more accessible to the solvent than Mn2 (Figure 3b) and is coordinated by two aspartic residues (D8 and D37), one glutamine (Q10), and three water molecules, whereas Mn2 is coordinated by D37 (bridging both ions), as well as N65, H120, H228, and two water molecules (Figure 3a zoom). The groove hosting the metal‐binding site is flanked by a flexible region and by a large rigid cleft on the opposite side (hereafter named as subsite 1 and subsite 2, respectively). In the former region, no interpretable electron density was observed for the residues 197–202, indicating a disordered loop (Figure 3a,b).

A noteworthy finding emerged from comparison of the *Pa*ApaH–Mn and *Pa*ApaH–Mg structures. Although the global fold is conserved, only a single Mg^2+^ ion could be modeled in the latter, occupying the Mn1 position. All coordinating residues adopt similar conformations between the two structures, except N65 (Figure 3c). In the Mn‐bound form, N65 coordinates Mn2, whereas in the Mg‐containing structure the metal ion occupying the Mn2 position is replaced by a water molecule, and N65 flips outward being no longer involved in metal coordination (Figure 3c). The lack of a second metal ion in the *Pa*ApaH–Mg structure may explain the protein inactivity in the presence of Mg^2+^ in the tested conditions.

**FIGURE 4:**
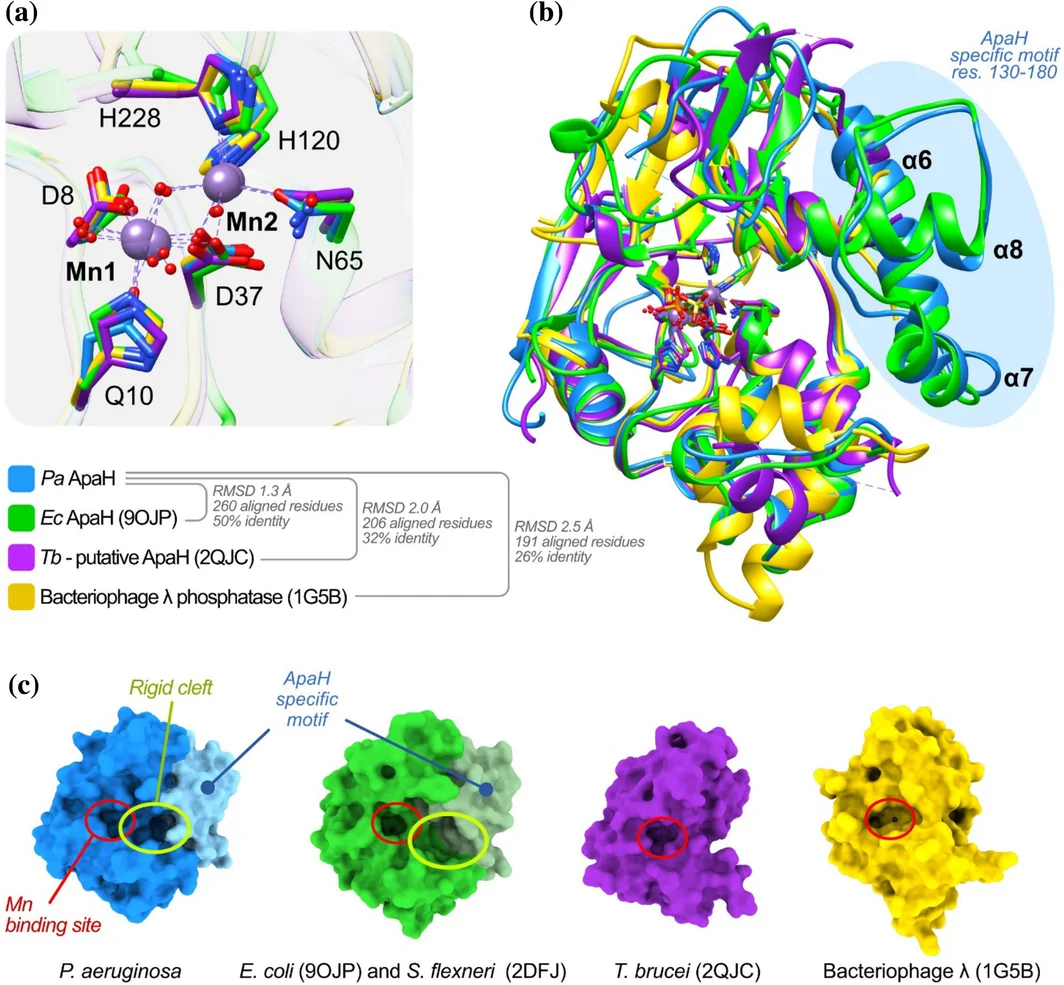
Structural comparison of *Pseudomonas aeruginosa* diadenosine tetraphosphatase (*Pa*ApaH) with related phosphatases. (a) Close‐up view of the binuclear metal center in *Pa*ApaH–Mn (blue sticks) superimposed with that of homologous structures from *Escherichia coli* (*Ec*ApaH: 9OJP (Nuthanakanti et al., 2025)—green sticks), *Trypanosoma brucei* (*Tb*ApaH: 2GJC (Almo et al., 2007)—magenta sticks) and bacteriophage λ (Ser/Thr protein phosphatase: 1G5B (Voegtli et al., 2000)—yellow sticks). Residues involved in metal coordination are shown as sticks and Mn^2+^ ions are represented as purple spheres. Coordination distances are shown with purple dashed lines. (b) Structural alignment of the same proteins as in (a) shown as ribbons. The light blue oval highlights the ApaH‐specific structural motif (residues 130–180) that is observed only in *Pa*ApaH and *Ec*ApaH. (c) Surface representations of *Pa*ApaH and its homologs colored according to the same color code in panels (a) and (b). The position of the metal‐binding sites is marked by a red circle, while the rigid cleft formed by the ApaH‐specific motif in *Pa* and *Ec/Sf*ApaH (lighter coloration) is marked by a green circle.

Distance‐matrix ALIgnment (DALI) search (Holm, 2022) identified *Pa*ApaH as closely structurally homologous to: (i) ApaH from *E. coli* or *S. flexneri* (respectively herein referred to as *Ec/Sf*ApaH, Z score: 39.7 and sequence identity 50%) (Nuthanakanti et al., 2025; Wang et al., 2006); (ii) a putative diadenosine tetraphosphatase from *Trypanosoma brucei* (*Z* score: 25.5 and sequence identity 32%) (Almo et al., 2007); (iii) a bacteriophage λ Ser/Thr protein phosphatase (*Z* score: 20.9 and sequence identity 26%) (Voegtli et al., 2000). The metal‐binding residues are conserved among these structures, except for Q10 of *Pa*ApaH which is replaced by a histidine in all the other three structures (Figures 4a and S2). The main difference is represented by a large insertion in *Pa*ApaH and *Ec/Sf*ApaH (residues 130–180) with respect to the others (Figures 4b and S2). This insertion corresponds to three mutually perpendicular alpha helices (α6–α7–α8) and an extended loop that in *Pa*ApaH and *Ec/Sf*ApaH contribute to shape the large rigid cleft flanking the metal‐binding site (Figure 4b,c).

### 2.3 ADP binds preferentially to the flexible region inducing folding of the disordered loop

**FIGURE 5:**
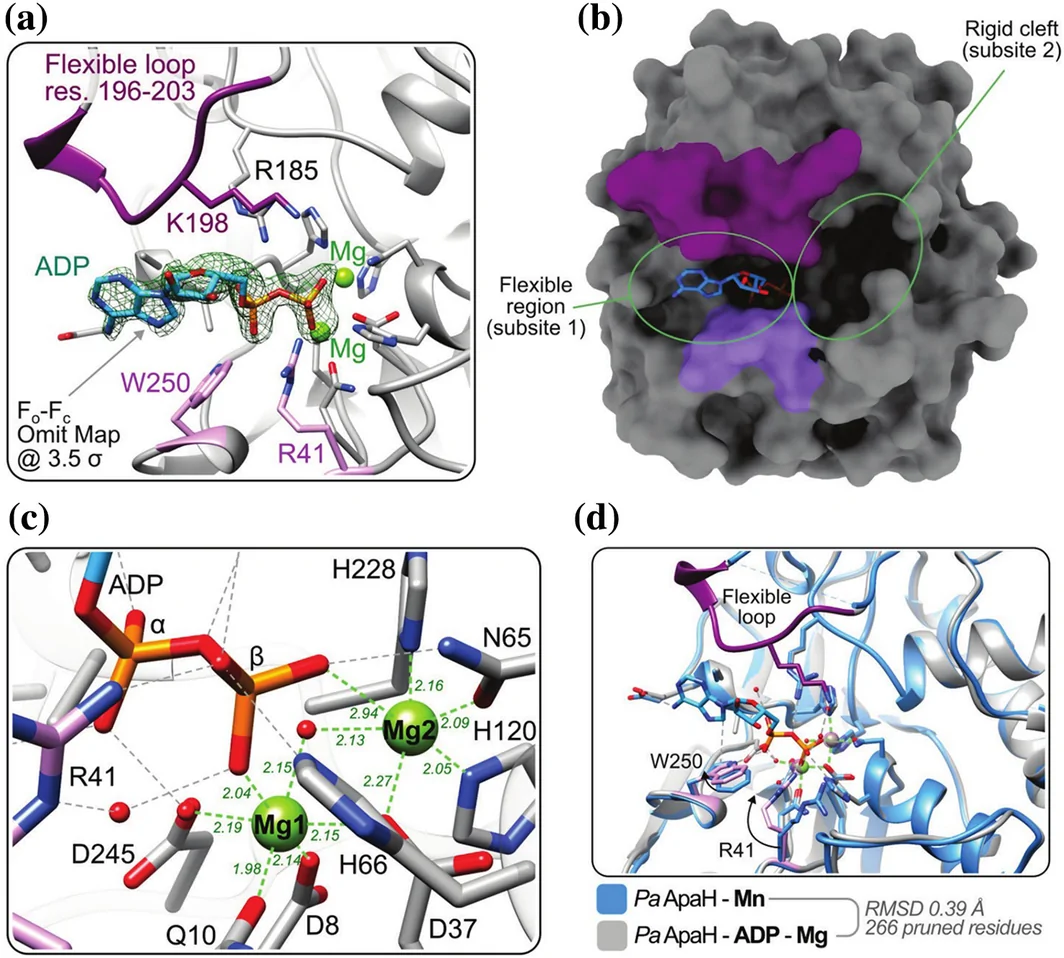
Structural basis for ADP binding to *Pseudomonas aeruginosa* diadenosine tetraphosphatase (*Pa*ApaH). (a) Close‐up view of the ADP·Mg^2+^ binding site in the *Pa*ApaH–Mg–ADP structure. The key residues interacting with ADP (cyan sticks) are represented as sticks. The *F*_o_‐*F*_c_ omit electron density map (contoured at 3.5*σ*) is shown for ADP. The residues belonging to the flexible loop (residues 196–203) that are now visible are shown in purple. The side chains of W250 and R41, that also interact with ADP on the opposite side of the flexible loop, are shown as light purple sticks. (b) Surface representation of the *Pa*ApaH–Mg–ADP structure. The rigid cleft (subsite 2) and the flexible region (subsite 1) are indicated by the green circles. ADP is represented as blue sticks. The region of the protein surface corresponding to the flexible loop is colored in purple while W250 and R41 are in light purple. (c) Detail of the coordination geometry of the ADP·Mg^2+^ complex. Mg1 and Mg2 are coordinated by conserved residues and the β‐phosphate groups, forming a distorted octahedral geometry. Hydrogen bonds and coordination distances (in Å) are indicated by green and gray dashed lines, respectively. (d) Structural superposition of the *Pa*ApaH–Mg–ADP (light purple) and *Pa*ApaH–Mn (blue) structures shown as ribbon. The root‐mean‐square deviation (RMSD) for the superposition is indicated below the panel. Binding of ADP induces a conformational change of the flexible loop and of the surrounding residues such as R41 and W250. The shift of R41 side chain conformation is indicated by a black arrow.

The *Pa*ApaH–Mg–ADP complex was obtained by soaking the apo crystals (grown in the presence of MgCl_2_) in a solution containing 50 mM ADP. The electron density map allowed us to model just one ADP molecule in the active site; however, the ADP‐bound *Pa*ApaH structure (Figure 5a) offers valuable insight into molecular determinants of substrate recognition. ADP binds to the flexible region (subsite 1) flanking the metal center hosting two Mg^2+^ ions (Figure S3). The β‐phosphate of ADP replaces two water molecules (one from each metal) out of the five metal‐coordinating ones observed in the apo *Pa*ApaH–Mn structure. Interestingly, ADP binds to *Pa*ApaH in the same position and in a similar conformation as that observed in the *Ec*ApaH‐ADP complex (PDB 9OJQ; Nuthanakanti et al., 2025) (Figure S4), while the rigid cleft (subsite 2) remains empty, indicating that ADP has a much lower affinity for this subsite than for subsite 1 (Figure 5b).

In the *Pa*ApaH–Mg–ADP, the flexible loop (residues 196–203), which is disordered in the apo *Pa*ApaH–Mn structure, can be modeled. This ligand‐induced conformational transition is likely driven by interactions of the K198 side chain, which forms H‐bonds with two water molecules interacting with the β‐phosphate group, as well as hydrophobic interactions with the ribose moiety (Figure S3). Additional hydrophobic interactions between the protein backbone and the nucleotide further stabilize the ADP‐protein interaction, thereby promoting structuring of the flexible loop (Figure 5a,d). The adenine base is also stabilized by a T‐shaped aromatic interaction with the conserved W250 residue and makes a sub‐optimal H‐bond (3.4 Å) with E233, which is not conserved among other ApaH from Gram‐negative bacteria (Figure S8). Finally, the β‐phosphate of ADP, which makes a bidentate coordination to both Mg^2+^ ions, is further interacting with R185 and R41 on opposite sides. Notably, R41 undergoes a conformational flip toward the β‐phosphate, contributing to its stabilization in a geometry suitable for nucleophilic attack by the activated hydroxyl ion coordinated by both metals (Figure 5c,d).

### 2.4 Computational analysis predicts the possible binding mode of Ap4A and other substrates

**FIGURE 6:**
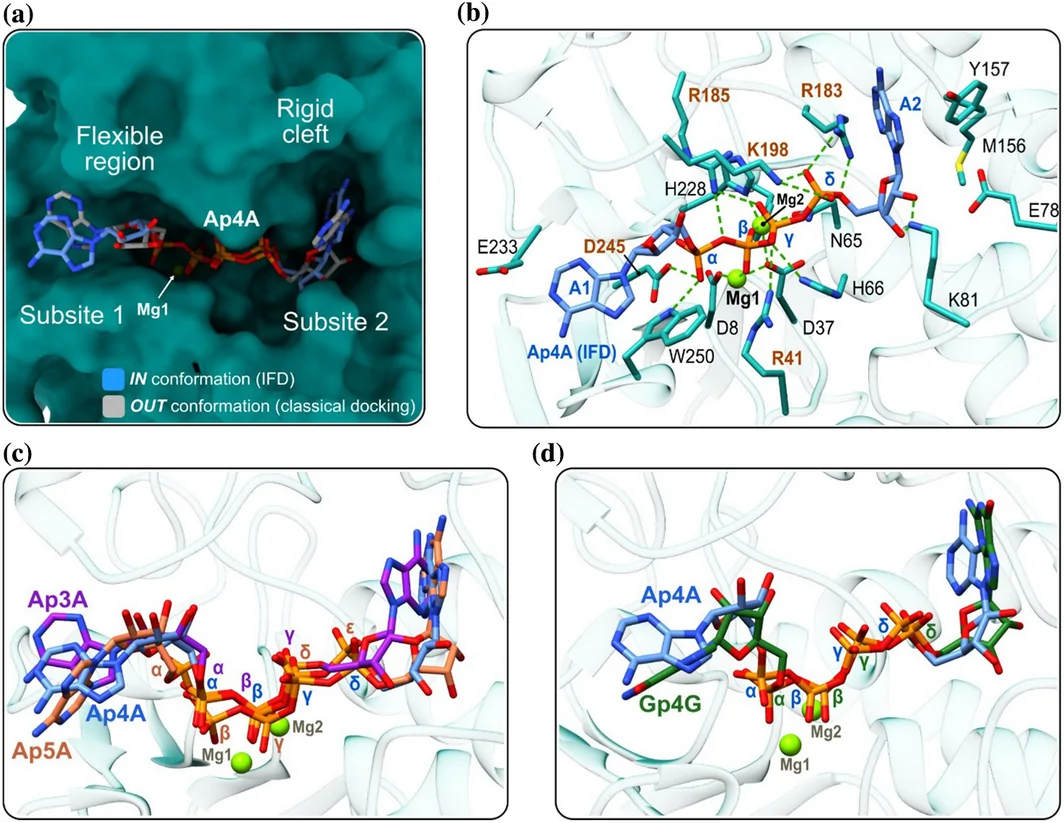
Docking analysis of diadenosine tetraphosphate (Ap4A) and other substrates. (a) Computationally predicted Ap4A binding poses to subsite 1 and subsite 2. The conformation obtained by classical docking prediction (*OUT*—light gray sticks) is superposed with the one obtained by induced fit docking (IFD) (*IN*—blue sticks). The protein is shown in dark green surface representation to better highlight the substrate binding groove. (b) Ap4A docking pose obtained by IFD. The side chains of the residues that shape the binding cleft and predicted to interact with Ap4A are shown as stick. The adenine base binding in subsite 1 is labeled A1, whereas the one binding in subsite 2 is labeled A2. H‐bonds interactions are shown as green dashed lines. The five residues that were selected for mutagenesis analysis (see below) are highlighted in orange. (c) Superposition of the proposed IFD conformations for Ap4A (blue), Ap3A (purple) and Ap5A (orange). (d) Superposition of the proposed IFD conformations for Ap4A (blue) and Gp4G (green).

Attempts to solve the crystallographic structure of *Pa*ApaH in complex with Ap4A were unsuccessful, likely due to the basal hydrolytic activity of the enzyme even in the absence of Mn^2+^. Therefore, to gain insight into the substrate binding conformations, we performed extensive computational docking simulations. Firstly, the method was validated by its capability to reproduce the ADP binding mode. Indeed, docking analysis resulted in ADP bound in subsite 1 with a conformation superposable to those observed in the crystal structures (Figure S5). Then, classical docking analysis was performed with Ap4A. However, since the apo *Pa*ApaH–Mn structures showed a certain degree of disorder with respect to the structure with ADP bound, an induced fit docking (IFD) analysis (Sherman et al., 2006) was also carried out to account for protein flexibility and to capture mutual ligand‐receptor conformational adjustments (Miller et al., 2021). Docked Ap4A molecules from both predictions are largely superposable spanning from subsite 1 to subsite 2 with nearly identical nucleotide conformation in the former, whereas the IFD proposed binding mode shows a major difference in subsite 2 where the nucleobase flips its orientation docking deeper into the bottom of the cleft (Figure 6a). The two conformations were designated as “*IN*” (IFD) and “*OUT*” (classical docking) and further inspected by molecular dynamics. The trajectories analyses showed that the *IN* conformations had lower RMSD values, with the nucleosides being less solvent‐exposed and having a higher number of contacts. In general, the *IN* conformations were predicted to be more reliable than the *OUT* ones (Figures 6b and S6).

Analysis of Ap4A binding mode showed that the α‐ and β‐phosphate groups are entirely superimposable with that of ADP in the *Pa*ApaH–Mg–ADP complex, as well as all the other interactions observed in subsite 1. The γ‐ and δ‐phosphates are predicted to make polar contacts with R41, H66, K81, R183, R185, and K198. The analysis also predicts that the nucleotide base binding to subsite 2 may engage in a π‐cation interaction with R183, and an aromatic contact with Y157 (Figure 6b).

Finally, IFD was used to predict the possible binding modes of the other sub‐optimal substrates: Ap3A and Ap5A. All dinucleotides showed a consistent, almost superimposable binding mode (Figure 6c). The main interactions were conserved, and the nucleosides in subsite 2 were always in the *IN* conformation (Figure 6c). Similar results were obtained from the analysis of docking with Gp4G (Figures 6d and S6), Gp3G, and Gp5G (Figure S7).

### 2.5 Functional analysis of catalytic‐site mutants *in* *vivo* and *in* *vitro* suggests that ApaH is a potential drug target

**FIGURE 7:**
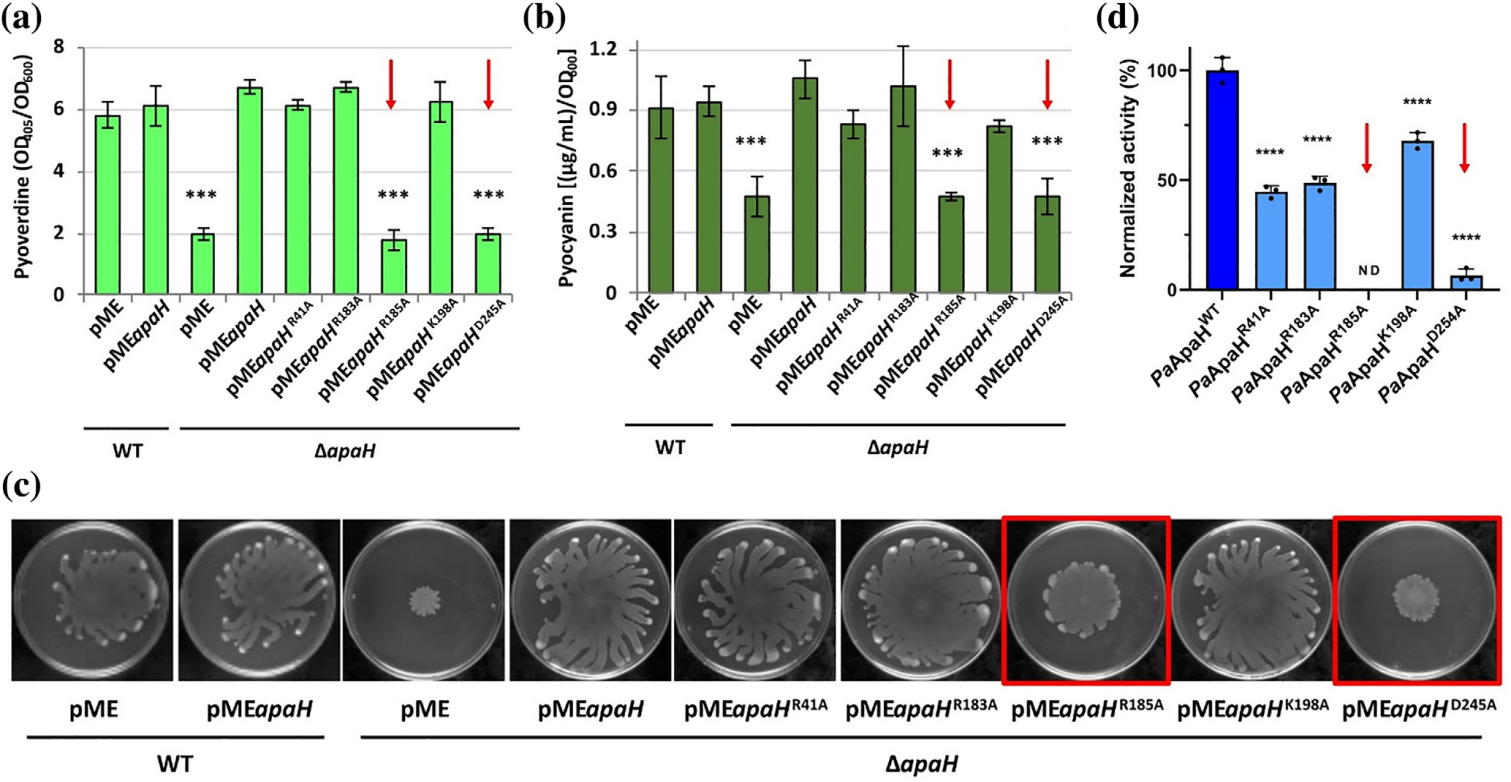
*In vivo* and *in vitro* characterization of *Pseudomonas aeruginosa* diadenosine tetraphosphatase (*Pa*ApaH) mutant variants. (a) Pyoverdine levels, (b) pyocyanin levels, and (c) swarming motility of *Pa* PAO1 wild‐type (WT) and the isogenic mutant Δ*apaH* carrying the empty plasmid pME6032 (pME) or its derivatives pME*apaH*, pME*apaH* ^R41A^, pME*apaH* ^R183A^, pME*apaH* ^R185A^, pME*apaH* ^K198A^, and pME*apaH* ^D245A^ as indicated. Values are the mean (± standard deviation [SD]) of three independent experiments. Asterisks indicate a statistically significant difference compared to Δ*apaH* carrying pME*apaH* (***p < 0.001; analysis of variance). Images in panel (c) are representative of three independent experiments giving similar results. (d) Hydrolysis of diadenosine tetraphosphate (Ap4A) by *Pa*ApaH mutants: Percentage activity with respect to the WT measured for the five variants by ion‐pair reversed‐phase high‐performance liquid chromatography in the same conditions as Figure 2c. Values are the mean (± SD) of three experiments. Statistical significance was determined using unpaired Student's *t*‐test (****p < 0.0001). Throughout the figure the results of the two variants R185A and D245A are highlighted in red. OD_405_, optical density at 405 nm; OD_600_, optical density at 600 nm.

Sequence alignment of bacterial ApaH homologs (Figure S8) revealed several highly conserved residues. Among these, residues R41, R183, R185, and K198 are predicted by our structural analysis to participate in substrate binding. Interestingly, D245, forming an H‐bond with a metal‐coordinating water, is also conserved despite not being directly implicated in substrate binding (Figure S3). To assess the functional relevance of these conserved residues, each of them was mutated to alanine. The corresponding *Pa*ApaH single mutant variants were expressed in a *Pa* mutant strain lacking the chromosomal *apaH* gene (PAO1 Δ*apaH*) and evaluated for their ability to restore some virulence‐related phenotypes regulated by *Pa*ApaH, namely pyoverdine and pyocyanin production and swarming motility. As shown in Figure 7a–c, expression of the protein variants R41A, K198A, and R183A restored all phenotypes at levels comparable to those of the wild‐type strain PAO1 or the Δ*apaH* mutant strain expressing the *Pa*ApaH wild‐type protein. In contrast, Δ*apaH* cells expressing the R185A or D245A variant of *Pa*ApaH displayed defects in both pyoverdine and pyocyanin production, as well as in swarming motility, comparable to the mutant strain carrying the empty plasmid, showing that these residues are crucial for *Pa*ApaH function in vivo. Notably, none of the mutant proteins had a negative impact on bacterial growth (Figure S9), implying that the inability of the R185A and D245A variants to restore virulence cannot be ascribed to a general toxic effect.

To confirm that the phenotypic defects of cells expressing the ApaH variants were due to impaired enzymatic activity of *Pa*ApaH, we recombinantly expressed the five variants in *E. coli*. All mutants were purified to homogeneity with a monodisperse elution profile obtained by SEC consistent with a monomeric state (Figure S10A). All variants are properly folded as shown by far‐UV CD analysis (not shown) with an apparent melting temperature (*T*_m,app_) comparable to that of the wild‐type protein (*T*_m,app_ ∽53.0°C) (Figure S10B). These results indicate that the mutations do not significantly affect protein folding or stability. Enzymatic assays carried out by Ion‐Pair RP‐HPLC revealed that, among the five mutants, *Pa*ApaH^R185A^ and *Pa*ApaH^D245A^ had the lowest catalytic activity. In particular, no catalytic activity was detectable for *Pa*ApaH^R185A^, while *Pa*ApaH^D245A^ retained only ~10% of wild‐type activity. The other three mutants (*Pa*ApaH^R41A^, *Pa*ApaH^R183A^, and *Pa*ApaH^K198A^) were also partially inactive but retained at least 45% of residual activity (Figure 7d).

These results indicate that an impaired catalytic activity of *Pa*ApaH correlates with virulence‐related markers affected by *Pa*ApaH.

## 3 DISCUSSION

In this study, we provide a comprehensive structural and functional characterization of *Pa*ApaH, defining its metal‐dependent catalytic activity and molecular aspect of substrate recognition. Importantly, we establish a direct link between its catalytic activity and expression of virulence traits in *Pa*.

At a biochemical level, *Pa*ApaH conforms to the canonical features of characterized ApaH homologs. Biochemical analysis revealed that *Pa*ApaH is similar to the homologs that have been characterized so far. It is a metal‐dependent Ap4A symmetrical hydrolase that cleaves the phosphoanhydride bond at the β–γ position, releasing two ADP molecules, with a *K*_M_ (3.6 ± 0.9 μM) for Ap4A comparable to those determined for ApaH homologs (Ismail et al., 2003; Nuthanakanti et al., 2025; Sasaki et al., 2014) and a moderate turnover (*K*_cat_ = 5.7 ± 0.3 s^−1^). *Pa*ApaH hydrolyzes other dinucleotide polyphosphates, or Np(n)Ns, with even lower efficiency, while remaining inactive toward mononucleotides (ADP, ATP, GDP, and GTP) in the tested conditions (Figures 2 and S1), highlighting a stringent substrate selectivity toward dinucleotides that likely prevents unwanted nucleotide turnover in vivo.

Structurally, *Pa*ApaH is monomeric in solution (Figure 1c), adopts a mixed α/β globular fold and belongs to the metallophosphoesterase (MPE) superfamily (Figures 1a and 3a) (Matange et al., 2015). As observed for other ApaH enzymes, catalysis relies on a bimetallic active site located in a surface groove, where two metal ions are coordinated by invariant residues (D8, D37, N65, H120, and H228), and by a glutamine (Q10) (Figure 3a) which is substituted by a histidine in the closest structural homologs (Figures 4a and S2) and in other Gram‐negative bacteria (Figure S8). Different water molecules also contribute to metal coordination. The overall fold of *Pa*ApaH is highly superposable with *Ec*ApaH (Nuthanakanti et al., 2025; Wang et al., 2006). Nevertheless, subtle but potentially functionally relevant differences are evident. In particular, *Pa*ApaH displays a disordered loop in subsite 1 of the nucleoside binding site which is structured in *Ec*ApaH. Furthermore, unlike *Ec*ApaH, the *Pa*ApaH–Mg structure reveals sub‐optimal metal coordination, resulting in an incomplete saturation of the bimetallic site. Residue N65, which coordinates Mn^2+^ at the M2 position in the *Pa*ApaH–Mn structure, undergoes a conformational rearrangement in the presence of Mg^2+^, losing its coordination (Figure 3c). Together with the distinct coordination chemistry of Mn^2+^ and Mg^2+^, these observations likely provide a structural rationale for the manganese dependence of *Pa*ApaH activity (Figure 1e). Notably, in the *Pa*ApaH–Mg–ADP structure, two Mg^2+^ ions occupy the bimetallic site, with N65 reverting to a coordinating conformation (Figure 5c). This suggests that ADP binding can partially compensate for the lower intrinsic affinity of Mg^2+^ for the M2 site. This is likely due to the presence of the negatively charged phosphate group that contributes to the Mg^2+^ stabilization.

**FIGURE 8:**
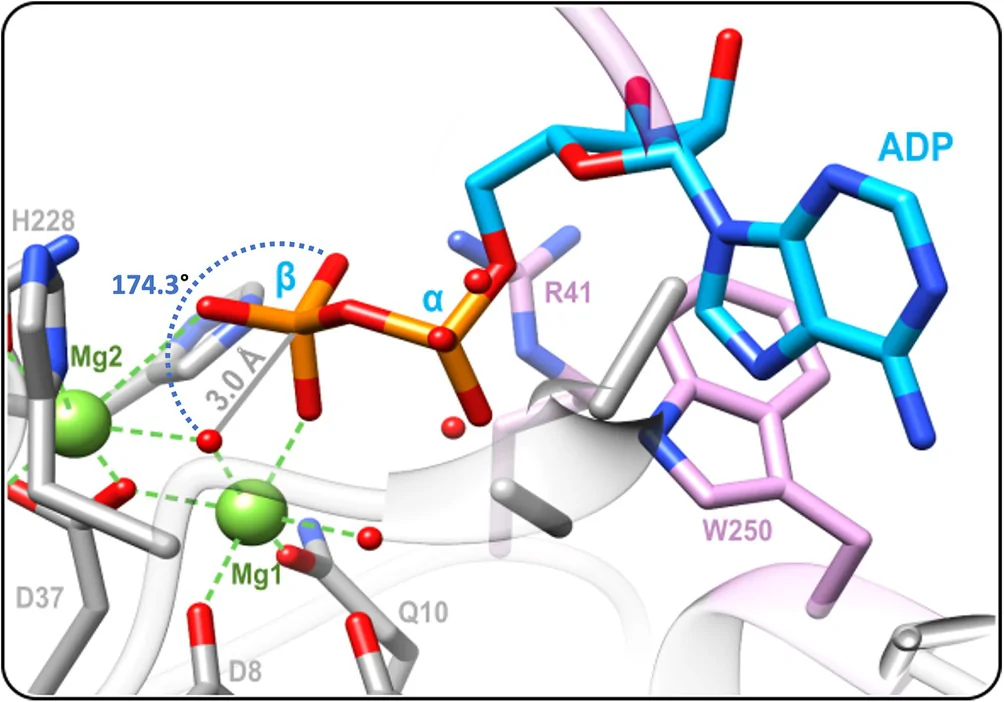
Position of the putative activated water in the *Pa*ApaH–Mg–ADP structure. Overview of *Pa*ApaH–Mg–ADP active site showing the position of a water molecule bridging the two Mg^2+^ ions (green spheres) and its relative distance from the β‐phosphate group of ADP (represented as cyan stick). The position and distances of this water molecule suggest a potential S_N_2 nucleophilic attack at the β‐phosphate of diadenosine tetraphosphate. Selected residues and interactions have been omitted for better visualization. *Pa*ApaH, *Pseudomonas aeruginosa* diadenosine tetraphosphatase (tetraphosphate hydrolase).

Although the catalytic mechanism of ApaH enzymes has not been experimentally resolved in full detail, structural and mechanistic similarities with the Ser/Thr protein phosphatase family (Figure 4) suggest a two‐metal ion mechanism. In this model, catalysis is proposed to proceed via an S_N_2‐like mechanism: one Mn^2+^ activates the substrate via Lewis acidity, while the second Mn^2+^ generates a nucleophilic hydroxide ion that attacks the activated β‐phosphorus atom, forming a transient pentacoordinate intermediate and leading to phosphoanhydride bond cleavage (Howard et al., 2015; Pecina et al., 2021; Schenk et al., 2012; Voegtli et al., 2000). Consistent with this model, the *Pa*ApaH–Mg–ADP structure (Figure 5a) reveals a water molecule bridging the two metal ions, ideally positioned for nucleophilic attack on the β‐phosphate (O‐P distance = 3.0 Å and O‐P‐O angle = 174.3°) (Figure 8). The β‐phosphate is extensively coordinated through electrostatic and H‐bond interactions involving conserved residues (R41, N65, H66, R185, and K198) (Figures S3 and S8), the two metal ions, and water molecules, forming an interaction network that locks the phosphate in a catalytically competent orientation (Figure 5c). These structural features are fully consistent with our biochemical data, which show cleavage between the β‐ and δ‐phosphate for all tested substrates (Ap4A, Ap3A, Ap5A, or Gp4G) (Figure 2). Although direct experimental evidence supporting this catalytic mechanism is lacking, similar conclusions were independently reached for *Ec*ApaH based on extensive structural analysis (Nuthanakanti et al., 2025). In this context, the *Pa*ApaH–Mg–ADP structure likely represents a snapshot of the catalytically competent conformation despite the presence of Mg^2+^. These data suggest that, although Mg^2+^ can support the formation of an enzyme–substrate‐like complex, its different chemical properties compared to Mn^2+^ may raise the energetic barrier for catalysis, explaining the absence of measurable hydrolytic activity under the explored experimental conditions.

Comparison with recently reported *Ec*ApaH structures further clarifies the molecular basis of substrate recognition. For *Ec*ApaH in complex with Np(n)Ns, only the electron density corresponding to one nucleoside and the α‐ and β‐phosphate groups are observed. The only visible nucleotide always occupies subsite 1, while the second nucleoside remains largely unresolved. Occasionally, as in the case of the *Ec*ApaH–Ap4A complex (PDB 9OLZ; Nuthanakanti et al., 2025), a partial density accounting for the δ‐phosphate was detected. Based on these observations and our data, we propose that ApaH contains two distinct binding regions: a high‐affinity subsite 1, optimized to bind a nucleoside‐diphosphate (NDP) in a conserved conformation, and a low‐affinity, more aspecific subsite 2, which accommodates the remaining portion of the different Np(n)N substrates. Molecular docking predicts that Ap4A spans the entire groove connecting the two subsites (Figure 6). In particular, the ADP moiety binding at subsite 1 was predicted to adopt an almost invariant pose that closely matches the crystallographic binding mode observed in *Ec*ApaH (Nuthanakanti et al., 2025) (Figure S5). Conversely, the second nucleotide displayed substantial conformational variability within subsite 2. Despite being computational predictions, these findings are consistent with available crystallographic evidence and provide a mechanistic explanation for substrate selectivity. Specifically, while subsite 2 contributes less to overall binding affinity, the requirement for a second nucleoside effectively prevents the hydrolysis of ATP and GTP, ensuring that they are not degraded at physiological concentrations. Consistently, *Pa*ApaH selectively hydrolyzes Np(n)Ns, displaying a preference for adenosine‐containing substrates, in agreement with recent reports on *Ec*ApaH (Nuthanakanti et al., 2025). Notably, the rigid cleft forming subsite 2 originates from three additional α‐helices (α6–α7–α8) that are absent in other related phosphatases (Figures 4 and S2), suggesting that this structural motif is a defining feature of ApaH enzymes and is critical for dinucleotide selectivity.

Sequence alignment of Gram‐negative bacterial ApaH homologs (Figure S8) identified a conserved signature. Based on this analysis and on our structural and computational results, we selected five conserved residues not directly involved in metal binding (R41, R183, R185, K198, and D245) for in vivo virulence‐related phenotypes analysis. We did not consider the metal‐binding residues as they are clearly critical for the activity as already demonstrated for *Ec*ApaH (Nuthanakanti et al., 2025). *Pa*ApaH mutant variants carrying the R185A or D245A substitutions failed to complement *Pa*ApaH‐dependent virulence phenotypes in the Δ*apaH* mutant, while Δ*apaH* clones expressing the R41A, R183A, or K198A variants were phenotypically similar to the wild‐type complemented Δ*apaH* strain (Figure 7a–c). Biochemical assays on the five purified variants showed a stringent correlation between the catalytic impairment and the virulence‐related phenotypes observed in vivo. In particular, R185 was essential for catalysis, as its substitution by an alanine completely abolishes enzymatic activity, while the *Pa*ApaH^D245A^ variant retains approximately 10% of wild‐type activity (Figure 7d). It is interesting to notice that a residual catalytic activity above 45%, observed for the other three variants, resulted in phenotypes indistinguishable from those of the strain expressing the wild‐type protein. Structural data of *Pa*ApaH in complex with ADP suggest that R185 stabilizes and orients the α‐ and β‐phosphates via electrostatic and H‐bond interactions. Given the suggested S_N_2 catalytic mechanism described above, the position of the β‐phosphate must be tightly controlled for the nucleophilic attack of the activated water molecule to occur (Figure 8). On the other hand, D245, although not directly involved in substrate binding, contributes to the active‐site organization by H‐bonding a water molecule coordinating the M1 metal ion as well as the α‐ and β‐phosphates of ADP (Figure S3). The two mutants R41A and R183A retained around 50% of the native activity. R41 is involved in ADP binding interacting with the β‐phosphate in the *Pa*ApaH‐ADP‐Mg structure (Figure S3), whereas R183 is predicted to stabilize the nucleobase in subsite 2. Given their residual activity, it is likely that these two residues do not directly affect the catalytic mechanism but only lower the substrate affinity. Finally, the mutant K198A displayed 75% residual activity. This residue is located in the flexible loop that becomes structured upon substrate binding. It makes an H‐bond with a water molecule interacting with the β‐phosphate. Its role is likely marginal, providing an additional positive charge and contributing to the stabilization of the polyphosphate chain. Our findings identify R185 and D245 as critical determinants of *Pa*ApaH function in *Pa*, and reinforce the key role of the high‐affinity subsite 1. In this region of the active site, small changes to the metal‐coordinating geometry or of the substrate binding conformation result in an inactive enzyme. On the contrary, an alteration of the structural properties of subsite 2, the more aspecific site, appears to impact substrate affinity.

Overall, our results establish for the first time a direct link between ApaH catalytic activity and virulence regulation in *Pa*. The combination of biochemical and phenotypic data demonstrates that enzymatic inactivation directly translates into a loss of virulence‐associated traits. Despite the detailed molecular pathway linking Ap4A levels to virulence is still largely unknown, our results on *Pa*ApaH mutants suggest that a threshold‐dependent signaling mechanism may be in place. If this on/off behavior is true, virulence traits should remain unaffected until Ap4A reaches a critical intracellular concentration triggering a rapid all‐or‐nothing cellular response, leading to repression of virulence traits. Given recent evidence implicating the *Ec*ApaH enzyme in mRNA decapping and stability control (Luciano et al., 2019; Nuthanakanti et al., 2025), it is tempting to speculate that impaired *Pa*ApaH activity may similarly affect RNA turnover in *Pa*, consistent with the pleiotropic effects associated with Ap4A imbalance in this bacterium (Cervoni et al., 2024). However, additional studies are needed to elucidate the molecular mechanisms underlying ApaH‐ and Ap4A‐mediated regulation of gene expression in *Pa*.

Notably, the absence of ApaH homologs in higher eukaryotes, its conservation across *Pa* strains (https://pseudomonas.com/), and its contribution to virulence in both reference and clinical *Pa* isolates (Cervoni et al., 2024) underscore the potential of *Pa*ApaH as a selective antibacterial target. Although experimental evidence supporting the druggability of *Pa*ApaH and its pharmacological validation is still lacking, our biochemical data, together with previous evidence demonstrating its contribution to *Pa* infectivity in animal models (Cervoni et al., 2024), support further investigation of *Pa*ApaH as a promising target for the development of antivirulence therapeutic strategies aimed at disarming *Pa* while minimizing the selective pressure for resistance associated with conventional antibiotics.

## 4 MATERIALS AND METHODS

### 4.1 Site‐specific mutagenesis and cloning procedures

Site‐specific mutated alleles of the *apaH* gene from *Pa* were generated as previously described (Cervoni et al., 2021) with few modifications. Briefly, polymerase chain reactions  were performed with the Q5 Hot Start High‐Fidelity DNA Polymerase (New England Biolabs) using 1 ng of pBS*apaH* (Table S1) as the template and specific primer pairs designed to replace the codon for the target amino acid with an alanine‐encoding codon (Table S2). These primers were designed using NEBaseChanger (http://nebasechanger.neb.com/) and manually edited. Each PCR amplicon was treated with the KLD Enzyme Mix (New England Biolabs) to phosphorylate and circularize the PCR product, and to digest the methylated plasmid (extracted from *E. coli*) used as the template. The samples were used to transform *E. coli* DH5αF’‐competent cells and clones carrying the mutated allele of interest were identified by colony PCR using the “check” primer for each mutation combined with the *apaH*_pME6032_RV primer (Table S2). The constructs carrying the mutated alleles were extracted and verified by DNA sequencing. Then, each mutated allele was excised from the corresponding pBS derivative by digestion with *Eco*RI and *Kpn*I and sub‐cloned into the shuttle vector pME6032 (Heeb et al., 2002) previously digested with the same enzymes, yielding the pME6032 derivatives for isopropyl β‐D‐1‐thiogalactopyranoside (IPTG)‐inducible expression of the ApaH mutant variants of interest in *Pa* (Table S1).

To generate the plasmid pET‐28*apaH*, the *apaH* coding sequence was amplified by PCR from the genomic DNA of *Pa* PAO1, using the Q5 Hot Start High‐Fidelity DNA Polymerase and the primer pair *apaH*_pET28_FW/*apaH*_pET28_RV (Table S2). The amplicon was digested with NdeI and HindIII, and directionally cloned into the pET28b vector previously digested with the same enzymes. The plasmids pET‐28*apaH* ^*R41A*^, pET‐28*apaH* ^*R183A*^, pET‐28*apaH* ^*R185A*^, pET‐28*apaH* ^*K198A*^, and pET‐28*apaH* ^*D245A*^ were generated using the same cloning strategy, except that pBS*apaH* ^*R41A*^, pBS*apaH* ^*R183A*^, pBS*apaH* ^*R185A*^, pBS*apaH* ^*K198A*^, and pBS*apaH* ^*D245A*^ (Table S1) were used as templates, respectively, along with the reverse primer *apaH* mut_pET_RV (Table S2). The pET28b derivatives were verified by restriction analysis and sequencing.

### 4.2 Proteins expression and purification

*E. coli* BL21 (DE3) cells were transformed with the recombinant vectors (pET‐28*apaH* or the pET‐28b derivatives carrying the mutated *apaH* alleles; Table S1) and grown at 37°C in Luria‐Bertani (LB) medium containing 30 μg mL^−1^ of kanamycin. For all variants, the same protocol of protein expression and purification was followed. Briefly, expression was induced at OD_600_ ≈0.9 with 0.3 mM IPTG; cells were grown overnight at 22°C, collected by centrifugation, frozen, and stored at −20°C. Cell paste was resuspended in Buffer A (20 mM Tris–HCl pH 7.4, 300 mM NaCl) containing one tablet of Complete Inhibitor Cocktail (Roche Diagnostic, Mannheim, Germany), ~0.5 mg mL^−1^ Lysozyme (Sigma‐Aldrich, St. Louis, MO, USA), 10 μg mL^−1^ DNase I (Sigma‐Aldrich, St. Louis, MO, USA), 1 mM phenylmethylsulfonyl fluoride (PMSF), and was sonicated and centrifuged. The soluble fraction was loaded onto a 5 mL HisTrap FF column (Cytiva, Marlborough, MA, USA) pre‐equilibrated with Buffer A with 20 mM imidazole and eluted by stepwise increase in imidazole concentration (from 50 to 500 mM). Fractions containing the protein were collected and buffer exchanged to Buffer A using a PD10 desalting column (Cytiva, Marlborough, MA, USA). His‐tag was removed by overnight proteolytic digestion at 4°C using 5 U mg^−1^ thrombin (Sigma‐Aldrich, St. Louis, MO, USA). After stopping digestion with 2 mM PMSF, the sample was loaded on a 5 mL HisTrap FF column. Digested protein was eluted with Buffer A containing 50 mM imidazole. Fractions containing the proteins were concentrated and subjected to SEC on a HiPrep 16/60 Sephacryl S‐200 HR column (GE Healthcare, Uppsala, Sweden) or on a Superdex 75 10/300 GL (GE Healthcare, Uppsala, Sweden) equilibrated with 20 mM Tris–HCl pH 7.4, 100 mM NaCl. A calibration curve for molecular weight estimation was generated using albumin (67.5 kDa), ovalbumin (43 kDa), chymotrypsinogen (25 kDa), ribonuclease A (13.7 kDa) as protein standards (GE Healthcare, Little Chalfont, UK). For all variants, protein concentration was determined spectroscopically using *ε*_280_ = 47,440 M^−1^ cm^−1^, as predicted by the Expasy ProtParam tool (Gasteiger et al., 2005). Proteins were concentrated up to 2–5 mg mL^−1^, flash frozen in liquid nitrogen, and stored at −20°C until use. The average yield was 10 mg L^−1^ for wild‐type *Pa*ApaH, 5 mg L^−1^ for *Pa*ApaH^K198A^ and *Pa*ApaH^D245A^; 2 mg L^−1^ for *Pa*ApaH^R41A^, *Pa*ApaH^R183A^, and *Pa*ApaH^R185A^. All the purified proteins contained a small fraction (<5%) of proteolyzed *Pa*ApaH (lacking the last 65 C‐terminus residues) as verified by mass spectrometry analysis for the wild‐type protein (data not shown), likely occurring during expression.

### 4.3 Circular dichroism spectroscopy

CD measurements were performed using a Jasco J‐815 CD spectrometer (Jasco Inc., Easton, MD, USA) equipped with a Peltier temperature control unit. Far‐UV static spectra were collected at 20°C using a quartz cuvette with a 0.1 cm path length (Hellma & GmbH & Co. KG, Müllheim, Germany). Spectra of 5 μM proteins, in 20 mM Tris–HCl pH 7.4, 100 mM NaCl, were acquired with a scanning speed of 100 nm min^−1^, a data integration time (D.I.T.) of 2 s and 2 nm band width; reported spectra represent the average of three independent acquisitions. Thermal denaturation profiles were recorded by monitoring ellipticity at 222 nm while increasing the temperature from 20 to 90°C (*T* increasing rate: 1°C min^−1^).

### 4.4 *Pa*ApaH pNPP phosphatase activity

The phosphatase activity of *Pa*ApaH was assessed spectrophotometrically by monitoring the hydrolysis of the pNPP (max λabs = 312 nm) into pNP (max λabs = 400 nm). Time‐course UV–vis spectra of pNPP (50 μM) in the presence of *Pa*ApaH (10 μM) were acquired at various time points (up to 3 h) using an Agilent 8453 diode array spectrophotometer (Agilent Technologies, Santa Clara, CA, USA) at 25°C in 20 mM Tris–HCl pH 7.4, 100 mM NaCl. Metal dependence of the *Pa*ApaH phosphatase activity was assessed using a SpectraMax Mini Multi‐mode Microplate Reader (Molecular Devices, San Jose, CA, USA) by incubating 1 μM *Pa*ApaH and 100 μM pNPP at 37°C for 3 h in 20 mM Tris–HCl pH 7.4, 100 mM NaCl and 1 mM of different salts (MnCl_2_, ZnCl_2_, CoCl_2_, MgCl_2_, CaCl_2_, CuCl_2_, NiCl_2_, FeSO_4_, or Fe_2_O_3_) individually added. The pH dependence of *Pa*ApaH phosphatase activity was carried out as described above in 100 mM buffers (Bis‐Tris: pH 6.5; Tris–HCl: pH 7.0, 7.4, 8.0, 8.5; Bicine: pH 9.0), 100 mM NaCl, 1 mM MnCl_2_. Control reactions (in each condition lacking *Pa*ApaH) were used to correct the background of pNPP and pNP.

### 4.5 *Pa*ApaH hydrolase activity

The Ap4A hydrolase activity of *Pa*ApaH was monitored by ion‐pair reversed‐phase high‐performance liquid chromatography (IP‐RP‐HPLC) using a Discovery® C18 column (5 μm, 25 cm × 4.6 mm; Supelco, Sigma‐Aldrich, St‐Louis, MO, USA) connected to a KNAUER Azura HPLC system equipped with an HT300A autosampler (HTA srl, Brescia, Italy). For enzymatic kinetics analysis, reactions were started by mixing equal volumes (200 μL) of enzyme and substrate solutions. Final concentrations were: *Pa*ApaH 5 nM; Ap4A (Jena Bioscience, Jena, Germany) 1, 2, 4, 5, 10, 20, 40, and 80 μM. The reactions were carried out at 20°C in 20 mM Tris–HCl pH 7.4, 100 mM NaCl, and 2 mM MnCl_2_. Aliquots of 120 μL were withdrawn after 1, 2, and 4 min of incubation, and the reaction was stopped by adding 40 μL of 100 mM (pH 8.0) ethylenediaminetetraacetic acid (EDTA) and removing the enzyme by heating at 95°C for 10 min and centrifugation. The supernatant was stored at −80°C until IP‐RP‐HPLC analysis. Time zero samples were prepared by mixing enzyme and substrate solutions directly with EDTA and following the same protocol as for the other samples. HPLC analysis was performed by injecting 100 μL of each sample in 100 mM KH_2_PO_4_/K_2_HPO_4_ (pH 5.8), 10 mM tetrabutylammonium bromide, and 8.5% acetonitrile at 1 mL min^−1^ flow rate. Eluted nucleotides were monitored by UV absorbance at 254 nm. Ap4A peak area was converted to the corresponding concentrations using a calibration curve obtained from standard Ap4A solution. Initial velocities were extracted from the linear phase of substrate consumption at each substrate concentration and fitted to the Michaelis–Menten equation to determine the kinetic parameters. All measurements were performed in triplicate.

To evaluate *Pa*ApaH substrate preference, Ap3A, Ap4A, Ap5A (Jena Bioscience, Jena, Germany), ADP, ATP, GDP (Sigma‐Aldrich, St. Louis, MO, USA), and GTP (GE Healthcare, Chicago, IL, USA) were used at a concentration of 20 μM as described above, with the only difference that the reactions were stopped after 10 min only by heating and centrifugation.

### 4.6 Crystallization and structure solution

Crystallization conditions were initially screened automatically using the Oryx 4 crystallization robot (Douglas Instruments Ltd., Hungerford, UK). Optimization of condition G10 from the Index screen (Hampton Research, Aliso Viejo, CA, USA) was performed by hanging drop vapor diffusion method at 21°C. All crystals were obtained by mixing equal volumes (1 μL) of protein solution (buffer: Tris–HCl 20 mM pH 7.4, NaCl 100 mM) and reservoir (26%–30% polyethylene glycol (PEG) 3350, 0.1M Bis‐Tris (pH 5.5); 0.2M MgCl_2_; 5% PEG 200). Crystals appeared in 3–5 days. The *Pa*ApaH–Mn and the *Pa*ApaH–Mg–ADP crystals were obtained by soaking crystals in mother liquor containing 1.7 mM Ap4A and 0.2M MnCl_2_ for 15 min or containing 50 mM ADP (soaking time 6 h), respectively. For data collection, crystals were flash‐frozen in liquid nitrogen and exposed to X‐ray at the ID23‐2 beamline of the European Synchrotron Radiation Facility synchrotron (Grenoble, FRA) or XRD2 beamline of Elettra synchrotron (Trieste, ITA). Data were processed with XDS (Kabsch, 2010) and scaled with AIMLESS (Evans & Murshudov, 2013) at 1.75, 1.89, and 1.55 Å final resolution for *Pa*ApaH–Mn, *Pa*ApaH–Mg, and *Pa*ApaH–Mg–ADP crystals, respectively. All crystals were obtained in the P2_1_2_1_2_1_ space group with two molecules in the asymmetric unit as estimated by Matthews coefficient (Liebschner et al., 2019). For *Pa*ApaH–Mn, initial phases were obtained by molecular replacement using the structure of *Sf*ApaH (PDB code: 2DFJ (Wang et al., 2006)) as search model in MOLREP (Vagin and Teplyakov, 1997) as implemented in the CCP4 suite (Agirre et al., 2023). For *Pa*ApaH–Mg and *Pa*ApaH–Mg–ADP, molecular replacement was performed using *Pa*ApaH–Mn as search model. Iterative cycles of model building and refinement were performed with Coot (Emsley & Cowtan, 2004) and Phenix (Liebschner et al., 2019). In the *Pa*ApaH–Mn structure, residues 197–202 and the last 12 residues could not be fitted in the electron density. In the *Pa*ApaH–Mg structure, residues 201–202 and the last 13 residues could not be fitted, whereas in the *Pa*ApaH–Mg–ADP, only the last 12 residues are lacking. For all structures, statistics for data collection, refinement, and model building are reported in Table 1.

The superposition of A and B independent chains for all the *Pa*ApaH structures yields RMSD values ranging between 0.23 and 0.34 Å across all pairs and, due to better electron density maps and lower *B*‐factors observed for the A chain of all three structures, chain A was chosen for structural analysis. Homologous structures were searched with DALI server (http://ekhidna2.biocenter.helsinki.fi/dali; Holm, 2022). Structure images, superposition, and analysis were produced or performed using UCSF Chimera (Pettersen et al., 2004). Sequence alignment was performed with Jalview (Waterhouse et al., 2009), and the corresponding image was created by ESPript3.0 (ENDscript—https://endscript.ibcp.fr; Robert & Gouet, 2014).

Coordinates and structure factors have been deposited in the Protein Data Bank (PDB) with the following accession codes: 9T9O (*Pa*ApaH–Mn), 9T9N (*Pa*ApaH–Mg), 9TA7 (*Pa*ApaH–Mg–ADP).

### 4.7 Docking

All the docking experiments were performed on a SuperMicro, Intel Xeon Silver powered machine (64 cores) running Ubuntu 20.04 LTS. The in‐house crystal structures of *Pa*ApaH–Mg–ADP were prepared using Maestro (Schrödinger Release 2022‐4: Maestro, Schrödinger, LLC, New York, NY, 2021) (Madhavi Sastry et al., 2013). Hydrogen atoms were added while water and other solvents were removed. The 3D structures of the substrates Ap3A, Ap4A, Ap5A, Gp3G, Gp4G, and Gp5G were drawn, protonated at physiological pH, and minimized using the OPLS4 force field included in the Maestro suite (Lu et al., 2021). Classical docking experiments were carried out by Glide (Yang et al., 2021). The IFD analyses were carried out by the Maestro module (Miller et al., 2021). It refined residues within 5 Å of the ligands while keeping fixed the position of residues involved in cation coordination and ADP binding: D8, Q10, H120, N65, R185, A230, and W250.

Molecular dynamics simulations of 100 ns were performed using Desmond (Schrödinger Release 2024‐4: Desmond Molecular Dynamics System, D. E. Shaw Research, New York, NY, 2024) (Bowers et al., 2006). The system was relaxed before the simulations with NVT and NPT protocols setting temperature to 300 K and pressure to 1 atm. Langevin thermostat and barostat methods were used. The molecular dynamics were performed three times to ensure reproducibility. Images shown in the manuscript were prepared with UCSF Chimera (Pettersen et al., 2004).

### 4.8 Phenotypic assays

*Pa* growth assays were performed in LB in 96‐well microtiter plates (200 μL of bacterial culture in each well) at 37°C in a Tecan Spark 10M microtiter plate reader. Growth was measured over time as the optical density at 600 nm (OD_600_) of the bacterial cultures.

Pyocyanin was extracted with 3 mL of chloroform from 5‐mL cell‐free supernatants of cultures grown for 20 h at 37°C in LB supplemented with 20 μM FeCl_3_ and then re‐extracted into 1 mL of 0.2 *N* HCl. The optical density at 520 nm (OD_520_) of the resulting solution was measured and multiplied by 17.072 to obtain the pyocyanin concentration in μg mL^−1^ (Essar et al., 1990), which was normalized to the OD_600_ of the corresponding bacterial culture.

Pyoverdine was measured as the optical density at 405 nm (OD_405_) of cell‐free supernatants obtained from bacterial cultures grown for 20 h in Casamino acids (CAA) medium (0.5% CAA, 0.4 mM MgCl_2_) (Visca et al., 1993) appropriately diluted in 100 mM Tris–HCl (pH 8.0), and then normalized to the OD_600_ of the corresponding bacterial culture (Imperi et al., 2009).

Swarming motility was assessed by spotting 5 μL of bacterial cultures in LB on swarming plates (0.5% bacteriological agar [Difco], 0.8% nutrient broth N. 2 [Oxoid], and 0.5% glucose) (Imperi et al., 2013). The plates were incubated for 16 h at 37°C and photographed with a ChemiDoc Imaging System (Bio‐Rad).

### 4.9 Statistical analysis

Statistical analyses of *in vivo* phenotypic assays and *in vitro* metal dependence assays were performed using the one‐way analysis of variance (ANOVA) followed by Tukey's multiple comparisons test. Unpaired Student's *t*‐test was used to evaluate the statistical significance of wild‐type *Pa*ApaH substrate turnover in substrate preference assays and of the enzymatic activity of *Pa*ApaH mutants relative to wild‐type protein. Significance was defined as *p* < 0.05 (*), *p* < 0.01 (**), *p* < 0.001 (***), *p* < 0.0001 (****). Results with *p* ≥ 0.05 were considered not significant (ns).

## AUTHOR CONTRIBUTIONS

**Gianluca Pistoia**: data curation, writing—review and editing, writing—original draft, investigation, formal analysis, visualization, funding acquisition, validation. **Matteo Cervoni**: investigation. **Flavia Catalano**: investigation. **Francesca Troilo**: investigation. **Francesca Guidi**: investigation. **Eleonora Comparini**: investigation. **Giuseppina Mignogna**: investigation. **Carlo Travaglini‐Allocatelli**: writing—review and editing. **Alessandro Giuffrè**: writing—review and editing, funding acquisition, resources. **Antonio Coluccia**: formal analysis, investigation, funding acquisition, writing—original draft, writing—review and editing, validation, supervision, data curation. **Francesco Imperi**: investigation, funding acquisition, writing—original draft, writing—review and editing, formal analysis, supervision, validation, data curation. **Adele Di Matteo**: conceptualization, investigation, funding acquisition, writing—original draft, writing—review and editing, formal analysis, project administration, data curation, supervision, visualization, validation. **Giorgio Giardina**: conceptualization, investigation, funding acquisition, writing—original draft, writing—review and editing, formal analysis, visualization, supervision, project administration, data curation, validation.

## Supporting information


**Data S1.** Supporting Information.

## Data Availability

The data that support the findings of this study are openly available in Protein Data Bank at https://www.rcsb.org/, reference number 9T9N; 9T9O; 9TA7.
