# Supplementary material for: Structural and functional insights into Pseudomonas aeruginosa ApaH, a diadenosine tetraphosphatase crucial for bacterial virulence
Source: Protein Sci. 2026 Aug 25;35(9):e70781. doi: 10.1002/pro.70781 (PMC13505596; doi:10.1002/pro.70781)
Supplement: Supplementary file 1 [file PRO-35-e70781-s001.docx]

**Supplementary Material**

**Structural and functional insights into *Pseudomonas aeruginosa* ApaH, a diadenosine tetraphosphatase crucial for bacterial virulence**

Gianluca Pistoia^1^, Matteo Cervoni^2^, Flavia Catalano^3^, Francesca Troilo^3^, Francesca Guidi^2^, Giuseppina Mignogna^1^, Carlo Travaglini-Allocatelli^1^, Alessandro Giuffrè^3^, Antonio Coluccia^4^, Francesco Imperi^2,5^, Adele Di Matteo^3*^, Giorgio Giardina^1*^

^1^Department of Biochemical Sciences "A. Rossi Fanelli", Sapienza University of Rome, Italy.

^2^Department of Science, University Roma Tre, Rome, Italy.

^3^Institute of Molecular Biology and Pathology, National Research Council, Rome, Italy.

^4^Department of Drug Chemistry and Technologies, Sapienza University of Rome, Rome, Italy.

^5^LIFE Institute for Research and Health Care - Santa Lucia IRCCS, Rome, Italy

(*) *These authors contributed equally.*

**Correspondence**:

Giorgio Giardina, Department of Biochemical Sciences "A. Rossi Fanelli", Sapienza University of Rome, Rome, Italy.

E-mail: [giorgio.giardina@uniroma1.it](mailto:giorgio.giardina@uniroma1.it)

Adele Di Matteo, Institute of Molecular Biology and Pathology, National Research Council, Rome, Italy

E-mail: [adele.dimatteo@cnr.it](mailto:adele.dimatteo@cnr.it)

**List of Supplementary Content**

**Table S1.** Plasmids used in this study.

**Table S2.** Primers used in this study.

**Figure S1.** *Pa*ApaH activity toward mononucleotides.

**Figure S2.** Dali-structural alignment.

**Figure S3.** LIGPLOT analysis.

**Figure S4.** Comparison of ADP binding mode in *Pa*ApaH and *Ec*ApaH.

**Figure S5.** Docking analysis of ADP binding conformation.

**Figure S6.** Docking and molecular dynamics simulations for Ap4A binding.

**Figure S7.** Docking results for Gp(n)G substrates.

**Figure S8.** Sequence alignment of ApaH from Gram-negative bacteria.

**Figure S9.** Growth curves of *P. aeruginosa* PAO1 (WT) and the isogenic mutants.

**Figure S10.** Biochemical characterization of recombinant *Pa*ApaH variants.

**Table S1.** Plasmids used in this study.

| **Plasmid** | **Relevant characteristics** | **Source of reference** |
| --- | --- | --- |
| pBluescript II (pBS) | Cloning and sequencing vector; ColE1 replicon; Ap^R^ | Stratagene |
| pBS*apaH* | pBS derivative carrying a 914-bp DNA fragment encompassing the apa*H* coding sequence | Cervoni et al. 2023 |
| pBS*apaH^R41A^* | pBS containing the *apaH* coding sequence with a mutation in the codon 41 leading to a R41A substitution | This work |
| pBS*apaH^R183A^* | pBS containing the *apaH* coding sequence with a mutation in the codon 183 leading to a R183A substitution | This work |
| pBS*apaH^R185A^* | pBS containing the *apaH* coding sequence with a mutation in the codon 185 leading to a R185A substitution | This work |
| pBS*apaH^K198A^* | pBS containing the *apaH* coding sequence with a mutation in the codon 198 leading to a K198A substitution | This work |
| pBS*apaH^D245A^* | pBS containing the *apaH* coding sequence with a mutation in the codon 245 leading to a D245A substitution | This work |
| pME6032 | IPTG-inducible expression vector; *lacI^Q^*, Tc^R^ | Heeb et al., 2002 |
| pME*apaH* | pME6032 derivative containing the *apaH* coding sequence downstream of the IPTG-inducible promoter | Cervoni et al. 2023 |
| pME*apaH^R41A^* | pME6032 derivative containing the *apaH^R41A^* coding sequence downstream of the IPTG-inducible promoter | This work |
| pME*apaH^R183A^* | pME6032 derivative containing the *apaH^R183A^* coding sequence downstream of the IPTG-inducible promoter | This work |
| pME*apaH^R185A^* | pME6032 derivative containing the *apaH^R185A^* coding sequence downstream of the IPTG-inducible promoter | This work |
| pME*apaH^K198A^* | pME6032 derivative containing the *apaH^K198A^* coding sequence downstream of the IPTG-inducible promoter | This work |
| pME*apaH^D245A^* | pME6032 derivative containing the *apaH^D245A^* coding sequence downstream of the IPTG-inducible promoter | This work |
| pET-28b | Vector for expression of N-terminally 6His-tagged proteins with a thrombin site | Novagen |
| pET-28*apaH* | pET-28b derivative carrying the apa*H* coding sequence in frame with the 6His tag | This work |
| pET-28*apaH^R41A^* | pET-28b derivative carrying the *apaH^R41A^* coding sequence in frame with the 6His tag | This work |
| pET-28*apaH^R183A^* | pET-28b derivative carrying the *apaH^R183A^* coding sequence in frame with the 6His tag | This work |
| pET-28*apaH^R185A^* | pET-28b derivative carrying the *apaH^R185A^* coding sequence in frame with the 6His tag | This work |
| pET-28*apaH^K198A^* | pET-28b derivative carrying the *apaH^K198A^* coding sequence in frame with the 6His tag | This work |
| pET-28*apaH^D245A^* | pET-28b derivative carrying the *apaH^D245A^* coding sequence in frame with the 6His tag | This work |

**Table S2.** Primers used in this study.

| **Primers** | **Sequence (5’-3’)^1^** | **Restriction sites^2^** | **Application** |
| --- | --- | --- | --- |
| *apaH*_pET28_FW  *apaH*_pET28_RV | ggaattccatATGGCGGTCTACGCGGTAG  cccaagctTCATGTCGGGGTACCTGTG | NdeI  HindIII | Generation of pET-28*apaH* |
| *apaH*_R41A_FW  *apaH*_R41A_RV  *apaH*_R41A_check_FW | gcgGGCCCGCAGTCCCTGG  GTTGACCAGGTCGCCGAC  GCGACCTGGTCAACgcg |  | Generation and validation of pBS*apaH^R41A^* |
| *apaH*_R183A_FW  *apaH*_R183A_RV  *apaH*_R183A_check_FW | gcgATGCGTTTCTGCACCGAAG  GGTGAAGTAGTTGGTGATCA  CACCAACTACTTCACCgcg |  | Generation and validation of pBS*apaH^R183A^* |
| *apaH*_R185A_FW  *apaH*_R185A_RV  *apaH*_R185A_check_FW | gcgTTCTGCACCGAAGATGGCA  CATGCGGGTGAAGTAGTTG  TACTTCACCCGCATGgcg |  | Generation and validation of pBS*apaH^R185A^* |
| *apaH*_K198A_FW  *apaH*_K198A_RV  *apaH*_K198A_check_FW | gctGAAGGCCTCGACACCGC  GCTCTTCAGGTCGAGCTTG  CTCGACCTGAAGAGCgct |  | Generation and validation of pBS*apaH^K198A^* |
| *apaH*_D245A_FW  *apaH*_D245A_RV  *apaH*_D245A_check_FW | gcgACCGGCTGCGTCTGGGG  CAGGGCGAACAGGCCGG  GCCTGTTCGCCCTGGcg |  | Generation and validation of pBS*apaH^D245A^* |
| *apaH* mut_pET_RV | cccaagctTACAGGGCGTCCACCGGA | HindIII | Generation of pET-28*apaH^R185A^* and pET-28*apaH^D245A^* |
| *apaH*_pME6032_RV | GGGGTACCTGTGTACAGGG | KpnI | Identification of clones carrying *apaH* mutated alleles |
| M13_FW  M13_RV  T7 | GTTTTCCCAGTCACGAC  AACAGCTATGACCATG  TAATACGACTCACTATAGGG |  | DNA sequencing |

^1^ Lowercase letters indicate the region of the primer that does not anneal to the template.

^2^ The restriction site used for cloning is underlined in the primer sequence.

**Figure S1. *Pa*ApaH activity toward mononucleotides**. Histograms represent substrate concentration before (dark color) and after 10 min incubation with *Pa*ApaH (light color). Reactions were carried out using 5 nM *Pa*ApaH and 20 μM of each substrate in 20 mM Tris-HCl (pH 7.4), 100 mM NaCl, 2 mM MnCl_2_. Error bar: mean ± SD. n = 3.

**Figure S2. DALI-structural alignment.** Pairwise DALI-structural alignment of *Pa*ApaH (P.aer – this work) closest homologues: ApaH from *E. coli* (E.col; PDB: 9OJP); ApaH from *S. flexneri* (S.fle; PDB: 2DFJ); *T. brucei* (T.bru; PDB: 2QJC); Ser/Thr Protein Phosphatase from Bacteriophage λ (BP λ; PDB: 1G5B). Numeration refers to *Pa*ApaH. (SS) refers to the secondary structure assignments (H/h: helix, E/e: strand, L/l: coil). The additional structural motif observed in *Pa*ApaH and *Ec/Sf*ApaH and the disordered loop observed only in *Pa*ApaH are boxed.

**Figure S3. LIGPLOT analysis**. Diagram of the active site interaction of the PaApaH-ADP-Mg structure predicted by LigPlot+ v.2.2.9^[1]^**.**

**Figure S4. Comparison of ADP binding mode in *Pa*ApaH and *Ec*ApaH.** Structural superposition between *Pa*ApaH-Mg-ADP complex (this work - grey) and *Ec*ApaH-Mn-ADP (9OJQ - green). Residues are numbered according to *Pa*ApaH.

**Figure S5. Docking analysis of ADP binding conformation.** *Pa*ApaH structure is depicted as sky blue cartoon. ADP from the *Pa*ApaH-Mg-ADP crystal structure is reported as grey stick, the ADP docking pose as cyan stick. Residues involved in direct contacts are reported as grey stick lines. H-bonds and coordination contacts are depicted as yellow dot lines.

**Figure S6. Docking and molecular dynamics simulations for Ap4A binding.** (**A**) *IN* conformation: Induced Fit Docking (IFD) in cyan, representative molecular dynamics snapshot in light grey. (**B**) *OUT* conformation: docking pose in cyan, representative molecular dynamics snapshot in gold. Below each panel the plot of the RMSD fluctuations of the ligands (red line) and proteins (gray line) for *IN* conformation (left) and *OUT* conformation (right) reported as a function of simulation time.

**Figure S7.** **Docking results for Gp(n)G substrates.** (**A**) Comparison of Gp4G binding conformation from classical docking (pink) and IFD (light green). The residues involved in interactions are reported as grey lines. Cations were reported as green spheres. H-bonds and polar contacts were depicted as dotted yellow lines. (**B**) Proposed IFD conformation for Gp4G (light green), Gp3G (blue) and Gp5G (white). Residues involved in interactions are also reported as stick.

**Figure S8. Sequence alignment of ApaH from Gram-negative bacteria.** Residues directly involved in metal binding are marked with a star. Substrate-coordinating residues, identified based on both crystallographic data and docking analyses, are highlighted with an orange Pac-Man symbol. Arrows indicate residues that were mutagenized for *in vivo* and *in vitro* analyses. Sequence alignment was performed with Jalview (Waterhouse et al., 2009) and image was created with ENDscript (Robert & Gouet, 2014).

**Figure S9.** **Growth curves of *P. aeruginosa* PAO1 (WT) and the isogenic mutants.** Growth curves of *P. aeruginosa* PAO1 (WT) and the isogenic mutant Δ*apaH* carrying the empty plasmid pME6032 (pME) or its derivatives pME*apaH*, pME*apaH*^R41A^, pME*apaH*^R183A^, pME*apaH*^R185A^, pME*apaH*^K198A^ or pME*apaH*^D245A^ as indicated, cultured in LB supplemented with 0.1 mM IPTG in 96-well microtiter plates at 37 °C in a TECAN Spark plate reader. Values represent the mean (± standard deviation) of three independent experiments.

**Figure S10. Biochemical characterization of recombinant *Pa*ApaH variants.** (**A**) Size-exclusion chromatography (SEC) profiles of *Pa*ApaH variants and WT in 20 mM Tris-HCl (pH 7.4), 100 mM NaCl (column: Superdex^TM^ 75 10/300 GL). (**B**) Normalized thermal denaturation profiles of *Pa*ApaH variants acquired by measuring the protein ellipticity at 222 nm; apparent melting temperatures (T*_m_*_,app_) were obtained by fitting the data to a sigmoidal curve. T_m,_*_app_* for the *Pa*ApaH variants are similar to that of the wild-type protein (T*_m_*_,app_ ∽ 53.0 °C).

1. Laskowski R A, Swindells M B (2011). LigPlot+: multiple ligand-protein interaction diagrams for drug discovery. J. Chem. Inf. Model., 51, 2778-2786. PMID:21919503; DOI:[10.1021/ci200227u](https://doi.org/10.1021/ci200227u)
